# Rv2577 of *Mycobacterium tuberculosis* Is a Virulence Factor With Dual Phosphatase and Phosphodiesterase Functions

**DOI:** 10.3389/fmicb.2020.570794

**Published:** 2020-10-22

**Authors:** Marina Andrea Forrellad, Federico Carlos Blanco, Rubén Marrero Diaz de Villegas, Cristina Lourdes Vázquez, Agustín Yaneff, Elizabeth Andrea García, Maximiliano Gabriel Gutierrez, Rosario Durán, Andrea Villarino, Fabiana Bigi

**Affiliations:** ^1^Instituto de Agrobiotecnología y Biología Molecular (IABIMO), Instituto Nacional de Tecnología Agropecuaria-Consejo Nacional de Investigaciones Científicas y Técnicas (INTA-CONICET), INTA, Buenos Aires, Argentina; ^2^Instituto de Investigaciones Farmacológicas (ININFA), Consejo Nacional de Investigaciones Científicas y Técnicas-Universidad de Buenos Aires (CONICET-UBA), Cuidad Autónoma de Buenos Aires, Argentina; ^3^Host-pathogen Interactions in Tuberculosis Laboratory, The Francis Crick Institute, London, United Kingdom; ^4^Unidad de Bioquímica y Proteómica Analítica (UBYPA), Instituto de Investigaciones Biológicas Clemente Estable & Institut Pasteur de Montevideo, Montevideo, Uruguay; ^5^Sección Bioquímica, Facultad de Ciencias, Universidad de la República (UdelaR), Montevideo, Uruguay

**Keywords:** *Mycobacterium tuberculosis*, virulence factor, Rv2577, phosphatase, phosphodiesterase

## Abstract

Tuberculosis, a lung disease caused by *Mycobacterium tuberculosis (Mtb)*, is one of the ten leading causes of death worldwide affecting mainly developing countries. *Mtb* can persist and survive inside infected cells through modulation of host antibacterial attack, i.e., by avoiding the maturation of phagosome containing mycobacteria to more acidic endosomal compartment. In addition, bacterial phosphatases play a central role in the interplay between host cells and *Mtb*. In this study, we characterized the Rv2577 of *Mtb* as a potential alkaline phosphatase/phosphodiesterase enzyme. By an *in vitro* kinetic assay, we demonstrated that purified Rv2577 expressed in *Mycobacterium smegmatis* displays both enzyme activities, as evidenced by using the artificial substrates *p-*NPP and bis-(*p-*NPP). In addition, a three-dimensional model of Rv2577 allowed us to define the catalytic amino acid residues of the active site, which were confirmed by site-directed mutagenesis and enzyme activity analysis, being characteristic of a member of the metallophosphatase superfamily. Finally, a mutation introduced in Rv2577 reduced the replication of *Mtb* in mouse organs and impaired the arrest of phagosomes containing mycobacteria in early endosomes; which indicates Rv2577 plays a role in *Mtb* virulence.

## Introduction

Tuberculosis (TB) is an infectious disease whose etiological agent is *Mycobacterium tuberculosis (Mtb)*. The last report of the World Health Organization (WHO) informed that about a quarter of the world’s population is infected with *Mtb*, with the consequent risk of developing TB, and that an estimate of 10 million of new cases and 1.45 million deaths occurred in 2018^1^. This treatable, and even curable, disease, which affects mainly developing countries, is one of the ten leading causes of death worldwide. To date, BCG is the available vaccine used for TB. However, BCG only prevents young people from developing the disease and fails to protect the adult population. Even when the total number of TB cases has remained practically unchanged in recent years, there has been a marked geographic imbalance in the number of new cases because of co-infection with HIV, the emergence of multi and extreme resistant strains to the drugs historically used and the poor socio-economic conditions of the infected population.

*Mtb* has evolved to persist and survive inside host macrophages by regulating the expression of certain virulence factors. These factors help the bacterium to adapt physiologically and metabolically to the hostile environment of the macrophage. One of the most efficient strategies displayed by *Mtb* to counteract the macrophage actions is to modulate the normal progression of the phagosome-containing mycobacteria, thus preventing it from maturing into an active phagolysosome. The modulation of the intracellular endosomal trafficking allows virulent mycobacteria to persist and replicate in a non-acidic niche and to avoid its immunological detection (Forrellad et al., 2013b). Mycobacterial phosphatases, together with complex lipids [such as mycolic acids, sulfolipids, phthiocerol dimycocerosate (PDIMs), trehalose-di and mono-mycolates, phosphatidyl-myo-inositol hexamannosides (PIM6)] and lipoglycans [such as mannose capped lipoarabinomannan (ManLAM)], play an essential role in this modulation (Jackson, 2014).

Phosphatases are enzymes that catalyze the hydrolysis of some phospho-substrates, including glycolysis intermediaries (fructose 1,6-biphosphate, phosphoenolpyruvate, glycerophosphate), phosphoproteins, trehalose-6-phosphate, phosphoinositol phosphate, nucleotides and nucleic acids. These enzymes are classified into two large groups according to their molecular mass: high molecular weight (HMW) of around 55 kDa and low molecular weight (LMW) of approximately 35 kDa. The better characterized LMW phosphatases are widely distributed in animals, plants, fungi and prokaryotes. The broad catalog of phosphatases in *Mtb* comprises protein tyrosine phosphatases (PTP), phosphoserine phosphatases, lipid phosphatases and phosphodiesterases (PDE). PtpA and PtpB are the unique members within PTP and the most studied phosphatases of *Mtb* (Bach et al., 2008; Beresford et al., 2009; Wong et al., 2011; Margenat et al., 2015). On the other hand, PstP and SerB2 (Boitel et al., 2003; Wehenkel et al., 2007; arora et al., 2014) are representatives of phosphoserine phosphatases, whereas SapM is a lipid phosphatase (Saleh and Belisle, 2000; Vergne et al., 2005; Fernandez-Soto et al., 2019) and CdnP and mPDE are PDE (Shenoy et al., 2005, 2007; Dey et al., 2017). The joint action of these enzymes is critical to the virulence of *Mtb*. Indeed, each enzyme targets specific molecules that generally lead to the inhibition of recruitment and binding of the lysosome to the phagosome containing the mycobacteria (Beresford et al., 2009; Puri et al., 2013; Zulauf et al., 2018).

Particularly, Rv2577 is annotated as a putative phosphatase (Cole et al., 1998) with a protein structure homologous to that of the eukaryotic purple acid phosphatase (PAP), mainly of plants (Schenk et al., 2001). A previous study by our group revealed that the expression of *Mb2608*, the ortholog of *Mtb Rv2577*, was upregulated, both *in vitro* cultures and inside bovine macrophages, in a hypervirulent *Mycobacterium bovis* strain, in relation to a more attenuated *M. bovis* strain (Blanco et al., 2009). Thus, *Mb2608* may participate in *M. bovis* virulence; however, the function of *Mb2608* and Rv2577 remains mostly unknown.

With this in mind, we performed a kinetic and structural characterization of Rv2577. Furthermore, we evaluated its role in *Mtb* intracellular trafficking as well as in *Mtb* replication in macrophages and a mouse model of infection, respectively.

## Results

### Rv2577 Is an HMW Alkaline Phosphatase and Phosphodiesterase Enzyme

The Rv2577 protein has been annotated as a conversed hypothetical protein of unknown function according to TB database^2^. A BLASTp analysis demonstrated that Rv2577 shares about 25% of identity with PAPs of the metallophosphatase superfamily (MPP) (Supplementary Figure 1), a family mainly from plants, although it is also present in animals and fungi. This is a diverse family of binuclear metallohydrolases that catalyze the hydrolysis of many activated phosphoric acid mono- and di-esters as well as anhydrides.

**FIGURE 1:**
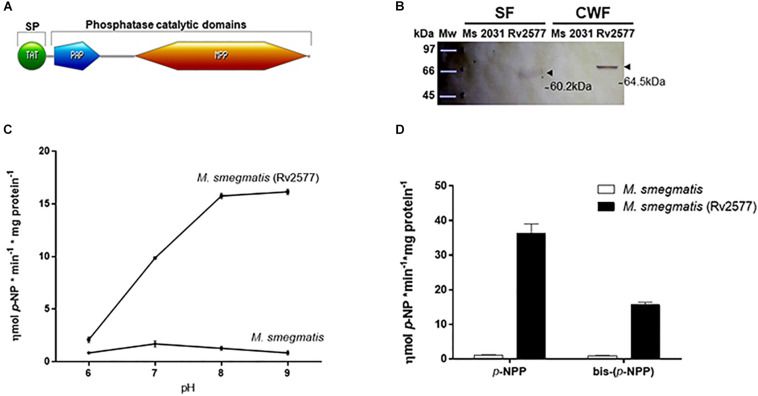
Rv2577 is an HMW alkaline phosphatase and phosphodiesterase enzyme detected mainly in the cell wall of mycobacteria. **(A)** Scheme of the Rv2577 primary sequence with its conserved domains: the Twin arginine translocation (TAT) signal peptide (SP) (1–52 residues), the purple acid phosphatase N-terminal domain (PAP, 67–150 residues) and the metallophos (MPP)/Calcineurin-like phosphoesterase domain (215–513 residues). According to Pfam database, these last two domains represent the phosphatase catalytic signature. **(B)** Western blot (WB) analysis with α-HA antibody showing the subcellular localization of Rv2577 in the soluble cytosolic and membrane (SF) or cell wall (CWF) fractions from *M. smegmatis* that overexpress the Rv2577-HA-His fusion protein (Rv2577). The same amount of total protein was loaded per lane, separated by SDS-PAGE and then transferred for WB analysis. The arrows indicate two reactive bands of ∼ 60.2 kDa (soluble isoform) and ∼ 64.5 kDa (cell wall isoform). *M. smegmatis* wild type (Ms) and *M. smegmatis* with the empty pML2031 vector (2013) were used as negative controls. **(C)** Phosphatase activity of Rv2577 in the soluble fraction from *M. smegmatis* wild type (*M. smegmatis*) and *M. smegmatis* overexpressing the Rv2577 protein [*M. smegmatis* (Rv2577)] at 6, 7, 8, or 9 pH with *p*-NPP as substrate.**(D)** Phosphatase and phosphodiesterase activities of IMAC-Ni^+2^-purified rRv2577 protein (black bars) and IMAC-Ni^+2^ purified proteins from *M. smegmatis* (white bars) at 8 pH with the artificial *p*-NPP or bis-(*p*-NPP) substrates, respectively. Graphs **(C,D)** represent the results obtained in three independent experiments performed in duplicate.

A PROSITE analysis identified a twin arginine translocation signal (TAT-signal) in the N-terminal of Rv2577 (first 52 amino acids) (Figure 1A). This finding suggests a cell wall location or secretion to the extracellular medium. In addition, a Pfam analysis demonstrated the presence of a PAP N-terminal domain (PF16656, residues 67–150) and a Metallophos MPP_PAPs or Calcineurin-like phosphoesterase domain (PF00149, residues 215–513) (Figure 1A). Both domains are present in metallophosphoesterases, such as phosphoserine phosphatases, 5′-nucleotidases, sphingomyelin phosphodiesterases, 2′,3′ and 3′,5′-cyclic-nucleotide phosphodiesterase as well as nucleases^3^

As mentioned above, the presence of a TAT signal peptide suggests the secretion of Rv2577 or its localization in the cell wall. Therefore, to investigate its subcellular location, we expressed Rv2577 as a Hemagglutinin-Histidine (HA-His) fusion protein (rRv2577) in the fast-growing mycobacterial *Mycobacterium smegmatis* (*M. smegmatis*-*Rv2577*). Curiously, according to a SDS-PAGE gel analysis, Rv2577 occurs in two isoforms with different apparent molecular weights: 64.5 kDa in the cell wall fraction and 60.2 kDa in the soluble fraction obtained in a buffer with detergent (Figure 1B and Supplementary Figure 2). A nano-HPLC-MS/MS analysis confirmed the presence of both Rv2577 isoforms in the gel bands. The results allowed the identification of 29 and 43 peptides corresponding to the Rv2577 sequence in the soluble (72% protein sequence coverage) and cell wall fraction (92% protein sequences coverage), respectively (Supplementary Table 1).

Even though the TAT signal sequence suggests the secretion of rRv2577, the WB and nano-HPLC-MS/MS analyses revealed no detectable rRv2577 protein in the culture supernatant of *M. smegmatis* (Supplementary Table 1 and ProteomeXchange with identifier PXD019769).

We also evaluated possible post-translational modifications that could account for the two apparent molecular weight forms in the cell wall fraction. The analyzed post-translational modifications included pupylation, phosphorylation and the most common glycosylation motifs previously reported in membrane and cell envelope proteins of *Mtb* (Birhanu et al., 2019). However, none of these modifications was detectable by nano-HPLC-MS/MS analysis (Supplementary Table 1. ProteomeXchange with identifier PXD019769).

Subsequently, we assessed *in vitro* the putative enzymatic activity of Rv2577 (soluble isoform) by evaluating its capability to hydrolyze artificial substrates. First, we determined that Rv2577 has an optimal alkaline pH for phosphatase activity (Figure 1C). This is similar to that of other reported prokaryotic PAPs (Yeung et al., 2009; Rodriguez et al., 2014) but different from that of eukaryotic PAPs, a family of enzymes with an optimal catalytic activity at 4–7 pH.

To further characterize Rv2577, we overexpressed rRv2577 in *M. smegmatis* and purified it by IMAC-Ni^+2^. The hydrolytic capability of rRv2577 against artificial substrates *p*-NPP and bis-(*p*-NPP) was 36.4 and 15.6 ηmol *p*-NP^∗^min^–1*^mg protein^–1^, respectively (Figure 1D). This result suggests that the phosphatase activity of Rv2577 is higher than its phosphodiesterase activity. This higher enzymatic activity was observed at physiological and alkaline pHs, regardless of the tested substrate (Supplementary Figure 3). According to a kinetic parameter analysis of rRv2577 (Table 1 and Supplementary Figure 4) the K_*m*_ of the specificity constant *k*_*cat*_/K_*m*_ was of a similar order for *p*-NPP (82μM) and bis-(*p*-NPP) (60μM), whereas the *k*_*cat*_ was 2.4 higher for *p*-NPP than for bis-(*p*-NPP) (Table 1). Thus, Rv2577 displayed a slightly higher *k*_*cat*_/K_*m*_ value for *p*-NPP.

**TABLE 1:** Kinetic parameters values of rRv2577.

Substrate	K_*m*_ (μm)	V_*max*_ (nmol *p*-NP.min^–1^.mg protien^–1^)	*k*_*cat*_ (min^–1^)	*k*_*cat*_/K_*m*_ (min^–1^ mM^–1^)
*p-*NPP	82	42	2.4	29.1
bis-(*p-*NPP)	60	17	1	16.8

*The reactions were carried out in the presence of rRv2577 87.4 ηM at 8 pH and 37°C by continuous method.*

Altogether, these data indicate that Rv2577 is a phosphatase, apparently an alkaline phosphatase, with the capacity to hydrolyze both phosphomonoester and phosphodiester substrates.

### The Structural Characteristics of Rv2577 Are Similar to Those of PAPs

**FIGURE 2:**
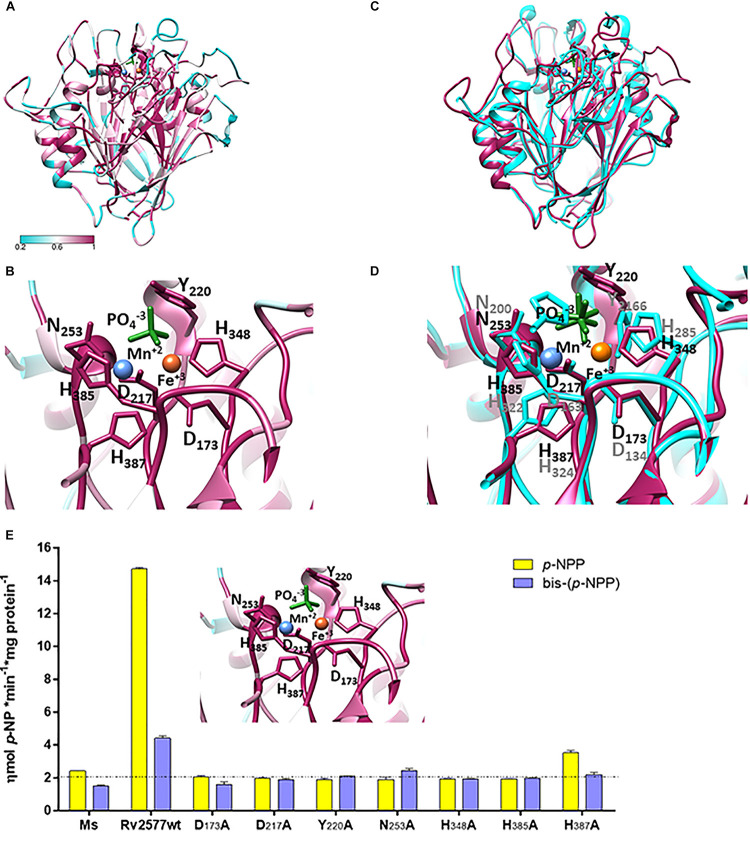
Rv2577 has a similar 3D structural model than that of the purpura acid phosphatases, with similar active site residues, as well, that are critical for catalysis. **(A)** The molecular model of Rv2577 based on sequences of 150 analogous proteins, including the top structure template of *Ipomoea batata* PAP (PDB 1XZW), alkaline prokaryotic and acid eukaryotic phosphatases. The color variation determines the degree of conservation of amino acid residues from minor (cyan) to major (magenta). **(B)** The catalytic site of Rv2577 is supported by residues D_173_, D_217_, Y_220_, N_253_, H_348_, H_385_ and H_387_ (represented as magenta sticks) and the divalent cations Mn^+2^ (blue ball) and Fe^+3^ (orange ball), which are part of the active site in 1XZW and are conserved in Rv2577. A phosphate group located in the active site is also represented (green sticks). **(C)** Overlapping structural model of Rv2577 (magenta) and 1XZW (cyan). **(D)** Amino acid residues that are part of the catalytic site based on **(C)**. **(E)** The proposed model (inset) was confirmed by replacement of each catalytic residue by alanine. Phosphatase and phosphodiesterase activities of the Rv2577 wild type (Rv2577wt) and Rv2577 mutants (D_173_A, D_217_A, Y_220_A, N_253_A, H_348_A, H_385_A and H_387_A) were determined against *p*-NPP (yellow bars) or bis-(*p*-NPP) (violet bars) as substrates. The dot line indicates the phosphatase and phosphodiesterase basal activities of soluble fraction of proteins from *M. smegmatis* wild type as negative control.

The three dimensional (3D) molecular modeling structure of Rv2577 was developed with the i-tasser server (Yang and Zhang, 2015). This analysis is based on multiple crystallographic structures of related phosphatase enzymes. The best model displayed a reasonable accuracy confidence score (C-score) of 0.7 (C-score is typically in the range of [−5, 2], where the higher value refers to a model with a high confidence and vice-versa) (Figure 2A). A PAP from sweet potato (PDB: 1XZW) was the most similar structural analog, with template modeling (TM) and root mean square deviation (RSMD) scores (Zhang and Skolnick, 2004) of 0.84 and 0.68, respectively. The estimation of the evolutionary conservation of residues in the protein was assessed with the ConSurf server (Ashkenazy et al., 2016) as a measure of their structural and functional importance.

Aligning the results from different experiments, we analyzed the sequence of 150 analogous proteins, including the top structure template PDB 1XZW, alkaline prokaryotic and acid eukaryotic phosphatase enzymes. The results identified a functional domain with highly conserved residues involved in the metal coordination and reaction mechanism (Figures 2A,B). Rv2577 presents a typical folding of PAPs and PDE, and maintains the typical consensus catalytic motif for the hydrolysis of phosphoesters. This consensus motif consists of seven invariable amino acid residues in five conserved domains: GDXX/GDXXY/GNH [D/E]/XXXH/GHXH (Richter, 2002). In Rv2577, particularly, this consensus is represented as GD_173_QS/GD_217_LCY_220_/GN_253_HE/VCMH_348_/GH_385_EH_387_. These residues would interact with the phospho-substrate and coordinate the bimetallic center Fe^+3^-Me ^+2^ (where Me as Fe ^+2^, Mn^+2^, Ca^+2^, or Mg^+2^ would be the typical cations). Functional and ligand analyses conducted by COACH predictor (within i-tasser; by comparing with interaction complexes at PDB) resulted in the prediction of two putative ligands: the divalent cation Mn and 1RF ligand and the oxyphosphorane analogs at PDBs 3RL4 and 6HWR, respectively (Figure 2B). Figure 2C shows the overlapped Rv2577 and 1XZW 3D models, whereas Figure 2D displays the similar arrangement of the amino acid residues that are part of their active sites.

Based on the structural information, we performed site-directed point mutations of D_173_, D_217_, Y_220_, N_253_, H_348_, H_385_ and H_387_ to determine the catalytic residues of Rv2577. These residues in the active site were replaced by the amino-acid alanine (Ala). All these mutants as well as the wild type rRv2577 were expressed as recombinant proteins in *M. smegmatis.* The wild type rRv2577 and mutants displayed similar levels of expression, thus indicating that Ala replacements did not affect protein stability (Supplementary Figure 5). We then evaluated the contribution of each amino acid residue to the hydrolytic activity against *p*-NPP and bis-(*p*-NPP). All of the mutations abrogated the hydrolytic activity of Rv2577 and resulted in an enzymatic activity similar to that observed for the negative control (Figure 2E).

These results demonstrate that the amino acids D_173_, D_217_, Y_220_, N_253_, H_348_, H_385_ and H_387_ are catalytic residues that form the active site of Rv2577; which suggests a catalytic mechanism similar to that of PAPs and confirms the proposed structure model.

### The Mutation in the *Rv2577* Gene Inhibits the Arrest of Macrophage Maturation

According to a previous study, the *Mb2608* gene, the orthologous of *Rv2577* in *M. bovis*, was overexpressed in a virulent *M. bovis* strain in relation to an attenuated *M. bovis* strain (Blanco et al., 2009). With this in mind, we evaluated the relevance of *Rv2577* in the replication of a pathogenic mycobacterium *in vivo* by using an *Mtb* CDC 1551 transposon mutant in the *MT2654* gene (the *Rv2577* ortholog) (Supplementary Table 2). The *Mtb* CDC 1551 wild type, the transposon mutant renamed as *Mtb* Rv2577 mutant (or Rv2577-mut) and the complemented strains were used to perform *in vivo* and *in vitro* experiments of virulence.

The interruption of *Rv2577* ortholog by transposon mutation carrying a kanamycin resistance, was confirmed by PCR analysis (Supplementary Figure 6). *Rv2577* does not seem to be part of an operon, since the surrounding genes in the genome are encoded in the opposite DNA strand to *Rv2577*. Therefore, the transposon insertion should not have produced any polar effect in neighboring genes. The *Mtb* Rv2577 mutant was complemented with an intact copy of Rv2577 gene cloned in the mycobacterial replicative plasmid pVV16 (Supplementary Table 2). The wild type, mutant and complemented strains displayed similar growth rates (Supplementary Figure 7), thus demonstrating that the transposon insertion did not impair the *in vitro* bacterial growth.

In addition, in accordance with reports in *M. bovis* (Blanco et al., 2009), Rv2577 gene was expressed during the *in vitro* growth of *Mtb* in rich media and intracellularly inside human THP-1 macrophages (Supplementary Figure 8).

One of the mechanisms whereby *Mtb* persists and survives inside the phagosome is by preventing the recruitment and fusion of lysosomes to infected phagosomes, thus inhibiting the bacterial lysis. Major phosphatases of *Mtb* such as PtpA, PtpB, and SapM block the binding between lysosome and the infected phagosomes (Wong et al., 2011, 2013; Puri et al., 2013; Zulauf et al., 2018).

**FIGURE 3:**
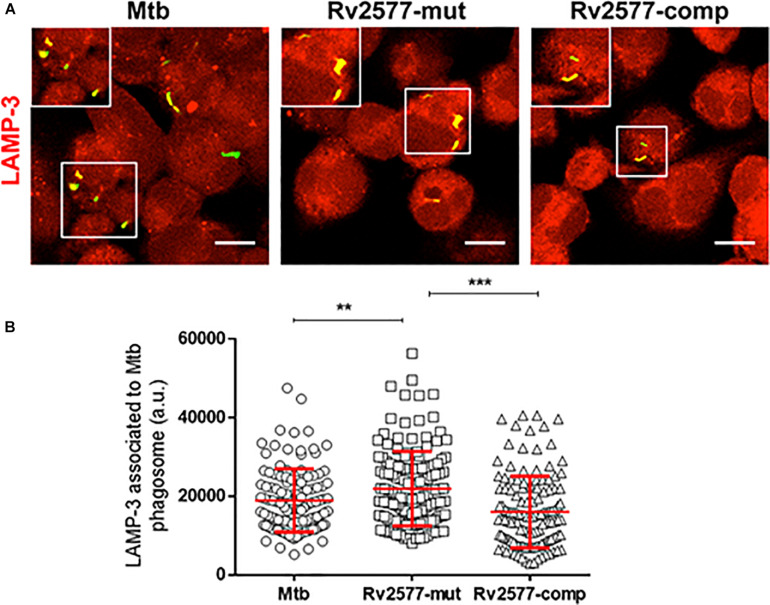
Rv2577 participates in the arrest of human phagosome maturation. **(A)** Human THP-1 macrophages were infected with *M. tuberculosis* wild type (Mtb), the Rv2577 mutant (Rv2577-mut) and the complemented (Rv2577-comp) strains to analyze the intracellular trafficking. The bacteria were labeled with FITC (green) and the LAMP-3 protein was detected using a specific antibody (red). **(B)** Quantification of the fluorescence observed in **(A)** (association of LAMP-3 to the mycobacterial compartment). The data are based on a representative experiment of three independent experiments, all performed in duplicate. ***p < 0.001, **p < 0.01 statistical analysis using ANOVA. Number of counted phagosomes = 150 from at least 50 different macrophages.

To evaluate the role of Rv2577 in the intracellular trafficking of *Mtb* in the human THP-1 macrophages, we quantified the association of the late endosomal marker LAMP-3 to the phagosome containing the *Mtb* wild type, Rv2577 mutant or complemented strains after 3 h of infection. Confocal microscopy and immunofluorescence analyses showed higher co-localization of Rv2577 mutant with LAMP-3 than the wild type and complemented strains (Figure 3). Similar impairment of phagosome arrest was detected for the Rv2577 mutant in the murine J774 macrophages, as evidenced by higher co-localization of the mutant to the late phagosome marker LAMP-2 (Supplementary Figure 9). However, no significant differences were detected between the intracellular survival of the three strains in human THP-1 macrophages (Supplementary Figure 10).

These results indicate that the *Rv2577* gene is expressed at early time of macrophage infection and suggest that the Rv2577 protein have a role in the modulation of phagosome maturation exerted by *Mtb* during macrophage infection.

### Rv2577 Is Relevant for *Mtb* Replication in Mice

**FIGURE 4:**
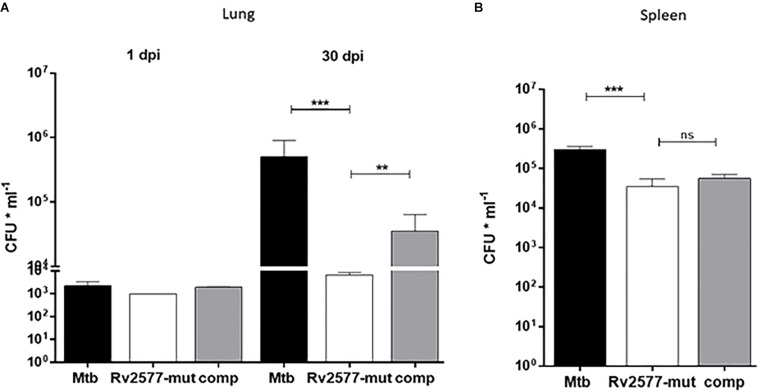
The Rv2577 mutation attenuates *M. tuberculosis* virulence in mice. The bacterial loads of *M. tuberculosis* wild type (Mtb), Rv2577 mutant (Rv2577-mut) and the complemented (Rv2577-comp) strains were measured as colony forming units (CFU*ml^– 1^) in the lungs **(A)** or spleen **(B)** of BALB/c mice at 1 and 30 days post-infection (dpi). All strains showed similar pulmonary bacillary load at 1dpi. The values are expressed as the mean ± SD. of CFUs for mouse organs (*n* = 6). ***p < 0.0001, **p < 0.001 calculated using ANOVA test.

An analysis of the bacterial loads of the *Mtb* wild type, Rv2577 mutant and complemented strains in BALB/c mice intratracheally infected with either of these strains revealed a significant decrease in the colony forming units (CFU) in lungs for the Rv2577 mutant, relative to the wild type or complemented strains, after 30 days post-infection (Figure 4A). Similarly, CFUs in the spleen of mice infected with the Rv2577 mutant were significantly reduced in comparison to the wild type (Figure 4B). However, the complemented strain failed to restore the wild type virulence, thus suggesting a possible loss of the replicative plasmid expressing Rv2577 during spleen infection. In fact, the growth of the complemented strain was strongly compromised in hygromycin containing media (resistance given by the plasmid) after infection. This finding suggests that the bacteria lost the plasmid by some still unknown mechanism. Likely, the absence of antibiotic in the *in vivo* environment impaired the selection of recombinant mycobacteria, thus affecting their survival capacity.

Altogether, Rv2577 mutant has an impaired replication in mice; which suggests that Rv2577 has a role in *Mtb* virulence.

## Discussion

In this study we detected two isoforms of Rv2577 expressed in *M. smegmatis*: one of low MW detected in the soluble fraction (which includes proteins from the inner membrane and the cytoplasm) and another, more abundant, of high MW, which reaches the cell wall. This result probably reflects the transit of Rv2577 from the cytosol, where it is synthesized, to the cell wall. The absence of this protein in the culture supernatant of *M. smegmatis* may indicate that Rv2577 is a protein anchoring to the wall, where it probably acts as a phosphatase/phosphodiesterase enzyme. In this line, mPDE expressed in *M. smegmatis* is mainly located in the cell wall (Chakraborti, 2016) and this location is promoted by the negative charges that confer phosphorylation by an eukaryote-like Ser/Thr kinases (Chakraborti, 2016; Malhotra et al., 2017).

Traini et al. (2017) have found that N-glycosylation occurs in the sphingomyelin phosphodiesterase acid-Like 3A (SMPDL3A), a member of Calcineurin-like phosphodiesterase. This modification is essential for protein secretion and for the inhibition of its degradation in the proteasome (Traini et al., 2017). We were unable to demonstrate any phosphorylation or glycosylation in Rv2577 by nano-HPLC-MS/MS analyses. Further characterization of Rv2577 is indispensable to understand the existence of the apparent two forms of this protein in the cell wall.

Rv2577 has both phosphatase and phosphodiesterase activities against artificial substrates. Unlike most eukaryotic PAPs (Schenk et al., 2005), although similarly to those of prokaryotic organisms (Shenoy et al., 2005; Yeung et al., 2009; Rodriguez et al., 2014), purified rRv2577 showed optimal activity at alkaline pH, with lower activity at acidic pH. In addition, the similar specificity constant *k*_cat_/K_m_ for *p*-NPP or bis-(*p*-NPP) suggests that the physiological substrate is a monophosphate ester or/and phosphodiester substrate. The K_m_ value of Rv2577 for *p*-NPP (80 μM), which can give an idea of the affinity for the substrate, was similar to that observed for the archetypical PAP 1xzw from *I. batatas* (K_m_ = 68 μM) (Schenk et al., 2005) and for PhoD from *Bacillus subtilis* (K_m_ = 50 μM)(Rodriguez et al., 2014). However, this parameter was four times lower than that of the PAP from *Burkholderia cenocepacia* (K_m_ = 330 μM) (Yeung et al., 2009). Regarding the V_max_ for *p*-NPP, all these enzymes showed a superior catalytic activity than Rv2577. The V_max_ of Rv2577 (V_max_ = 0,042 μmol *p*-NP^∗^min^–1*^mg protein^–1^), on the other hand, was similar to that reported for mPDE from *Mtb* (V_max_ = 0,017 μmol *p*-NP^∗^min^–1*^mg protein^–1^) (Shenoy et al., 2005). In contrast to Rv2577, mPDE has a clear better activity against bis-(*p*-NPP) (Shenoy et al., 2005), with a specificity constant 10 times higher than the value determined for Rv2577 (k_cat_/K_m_ = 0,192 min^–1^μM^–1^ vs. 0,017 min^–1^μM^–1^) (Shenoy et al., 2005). However, it is important to note that Rv2577 has a better affinity for bis-(*p*-NPP) (60 μM) than mPDE (K_m_ 1,300 μM).

These values could suggest that, in the contexts of the bacterium, Rv2577 acts when a low concentrations of phosphodiester substrates is presented in the medium, whereas mPDE becomes the best enzyme when the concentration of this substrate is increased.

Regarding physiological substrates, mPDE is more active toward phosphodiester compounds as the second messengers 3′,5′-cyclic AMP and cyclic-GMP (Shenoy et al., 2005) and 2′,3′-cAMP (Keppetipola and Shuman, 2008). Interestingly, the *Mtb* genome contains 13 genes for adenylyl cyclases, being mPDE the unique PDE that allows the maintenance of a correct cAMP homeostasis in the bacterium (Johnson and McDonough, 2018). In addition, YmdB from *B. subtilis* has the same structure domain and kinetic characteristic than mPDE and both enzymes share the same catalytic activity toward 3′,5′-cyclic AMP/cGMP and their preference for 2′,3′-cAMP and 2′,3′-cGMP compounds (Diethmaier et al., 2014). Thus, we can hypothesize that Rv2577 has a similar action against 3′,5′-cyclic AMP or cyclic-GMP substrates and that it fulfills a role in cAMP homeostasis. However, further studies are essential to assess this putative role, as the demonstrated activity toward the evaluated artificial substrates has not allowed us to conclude the precise physiological substrates of the enzyme. Thus, we cannot rule out that Rv2577 acts on other types of phosphorylated substrates, since a calcineurin-like phosphoesterase domain is also present in a large superfamily. This superfamily includes PAPs (Schenk et al., 2005; Yeung et al., 2009), PDEs (Shenoy et al., 2005; Diethmaier et al., 2014; Rodriguez et al., 2014), phosphoprotein phosphatases (PPPs), Mre11-SbcD DNA repair enzymes (Williams et al., 2008), Dbr1-like RNA debranching enzymes (Wang et al., 2013), YbbF-like UDP-2,3-diacylglucosamine hydrolases and acid sphingomyelinases (ASMases) (Flores-Díaz et al., 2016). The variety of substrates observed in this superfamily suggests that Rv2577 has a wide range of possible physiological substrates.

Rv2577 conserves the catalytic site structure of PAPs, which is similar to that of PDEs: the octahedral architecture of aspartic, histidine and asparagine in their active site. This suggests a similar catalytic mechanism with a binuclear metal center essential for catalysis (Richter, 2002). Mutations in any of the active site residues completely abrogated the *in vitro* enzymatic activities of Rv2577. According to this finding and to the molecular model of Rv2577, the residues D_173_, D_217_, Y_220_, and H_387_ in the active site could coordinate the iron metal, whereas the residues N_253_, H_348_ and H_385_ could coordinate manganese and finally the D_217_ residue could coordinate the oxygen Fe – O – Mn bridge.

Furthermore, the molecular model of Rv2577 allows us to hypothesize that Rv2577 has a binuclear active center similar to the archetypical PAP 1xzw from *I. batatas* (Schenk et al., 2005) or the PDEs mPDE from *Mtb* (Shenoy et al., 2007) and YmdB from *B. subtilis* (Diethmaier et al., 2014). However, Rv2577 would be different from the PAP PhoD from *B. subtilis*, which has an active site consisting of three metal, one iron and two calcium atoms (Rodriguez et al., 2014). This is because the residues D_265_ and N_271_-N_272_, which coordinate the second calcium atom in the PhoD active site, are not equally conserved in Rv2577; instead they have the G_252_, V_258_, and G_259_ amino acid residues.

Even when PAPs and PDEs share the same active site architecture, the presence of a tyrosine is typical of PAPs, such as 1xzw (Schenk et al., 2005) and PhoD (Rodriguez et al., 2014). This amino acid, by contrast, is absent in PDEs (Richter, 2002) such as mPDE (Shenoy et al., 2007) and YmdB (Diethmaier et al., 2014). Indeed, the Y_220_ residue in the active site of Rv2577 suggests that monophosphoesters are better target substrates for this enzyme.

In this study, Rv2577 showed to be essential for the full replication of *Mtb* in mice, at least in the infection model herein evaluated. As reported for several *Mtb* phosphatases such as PtpA, PtpB, and SapM (Singh et al., 2003; Beresford et al., 2007; Wong et al., 2011; Wong et al., 2013; Forrellad et al., 2013b), the contribution of Rv2577 to the *in vivo* persistence of *Mtb* could be, in part, to favor the arrest of phagosomes containing mycobacteria in non-lytic and non-acidic endosomal compartments. Rv2577 could be active in these environment conditions, as evidenced by its better *in vitro* catalytic activity at physiological and alkaline pHs.

However, the role of phosphatases in the virulence mechanisms of *Mtb* does not seem to be only limited to modulating phagosomal traffic. In this sense, PtpB promotes the decrease of pERK1/2 and p38 phosphorylated proteins, thus leading to lower levels of IL-6, iNOS suppression and IL-1β expression. All this, in turn, leads to a decrease in the inflammatory response of the host. In addition, PtpB increases phosphorylated Akt, which decreases the apoptotic activity of the macrophage (Wong et al., 2013; Fan et al., 2018).

On the other hand, a recent study has reported that PtpA, the most characterized *Mtb* phosphatase, regulates host gene transcription by binding to specific nuclear DNA regions (Wang et al., 2017). In the case of PDE, CdnP has a dual action that allows the maintenance of adequate levels of cAMP in the bacterium and cGAMP in the host. This leads to inhibition of the bactericidal response mediated by type I IFN and the activation of STING (Dey et al., 2017). This finding uncovers novel aspects of phosphatases and PDE activities in mycobacteria, and emphasizes the importance of these enzymes in the host-pathogen interactions of tuberculosis infections.

In summary, in this study we demonstrated that Rv2577 is relevant for the replication of *Mtb* in mice possibly because of its phosphatase and phosphodiesterase activities. Rv2577 is the first high molecular weight enzyme reported in *Mtb* with both phosphatase and phosphodiesterase activities *in vitro.* Because of this, we suggest a new name for Rv2577: *mapH*/MapH for mycobacterial alkaline phosphatase/phosphodiesterase.

Further studies involving the assessment of potential natural substrates of Rv2577 are necessary to better understand the role of this enzyme in the physiology of *Mtb* and in its participation in the interaction of the bacteria with the host.

## Materials and Methods

### Bacterial Strains and Culture Media

*M. tuberculosis* CDC 1551 wild type (*Mtb*), Rv2577 mutant (Rv2577-mut) and the complemented (Rv2577-comp) strains were grown in Middlebrook 7H9 medium (Difco Laboratories) supplemented with albumin 0.5%, dextrose 0.4%, Tween 80 0.05% and glycerol 0.5% (7H9-AD-T-G) or Middlebrook 7H10 (Difco Laboratories) supplemented with AD-T-G (7H10-AD-T-G). *M. smegmatis strain* ATCC 700084 mc^2^155 was grown in 7H9 or 7H10 medium supplemented with pyruvic acid sodium salt 0.4% (P) and tyloxapol 0.05%. Albumin was omitted from the media when preparing cultures for protein expression and purification. Antibiotics were added to the media as follows: 50 μg/ml hygromycin and 20 μg/ml kanamycin for mycobacterial growth or 200 μg/ml hygromycin, 100 μg/ml ampicillin and 50 μg/ml kanamycin for *E. coli* growth.

BEI Resources kindly donated the strains: *Mtb* CDC 1551 wild type and the *Mtb* CDC 1551 MT2654 mutant in the MT2654 gene, the ortholog to *Rv2577* gene in *Mtb* H37Rv. This mutant strain was named Rv2577 mutant or Rv2577-mut, since it is the same gene (Supplementary Table 2). Rv2577-mut is an *Mtb* CDC 1551 background strain with a Himar1 transposon insertion in the *MT2654* gene (the *Rv2577* gene ortholog). The lack of the intact *Rv2577* gene ortholog in Rv2577-mut was confirmed by PCR using the nptII (CAGACAATCGGCTGCTCTGAT) and low nptII (TGCGATGTTTCGCTTGGTGGT) set of primers to detect the Km^*R*^ insertion and FwRv2577Ms and RevRv2577Ms set of primers (Supplementary Table 2) to detect the Rv2577 wild type gene. The *Mtb* Rv2577 mutant was complemented with the replicative pVV16-Rv2577 plasmid carrying the *Rv2577* gene amplified from the *Mtb* CDC 1551 chromosome with the FwRv2577 (5′CATATGGTGGGCGCCGATCTG3′) and RevRv2577 (5′CATATGTATCCGCCGCGCGGC3′) set of primers and cloned in a *Nde*I restriction sites into the pVV16 vector (Supplementary Table 2).

The *in vivo* virulence experiments in mice, the *ex vivo* experiments in macrophages and cellular trafficking assays were performed with the *Mtb* strains cultured up to late exponential growth phase. The bacteria were harvested at 4,000 rpm for 10 min, washed and suspended in phosphate buffer saline (PBS). The bacterial suspensions were passed through a syringe needle (25 gauge) 30 times to disaggregate bacterial clumps, and then centrifuged at 1,000 rpm for 8 min to remove the clumps. The OD_600 *nm*_ was used as measure of bacterial density, where OD = 0.1 represents 1^∗^10^7^bacterium/ml, to subsequently calculate the multiplicity of infection (MOI). Infective doses were determined by bacterial counting in a Neubauer camera before BacLight staining.

### Expression and Purification of Rv2577 Enzyme

The *Rv2577* gene was amplified by PCR from the *Mtb* chromosome using the FwRv2577Ms and RevRv2577Ms set of primers (Supplementary Table 2). A fragment band of 1590 bp was purified from the agarose gel and cloned into pGEM^®^-T Easy vector (Promega). Recombinant pGEM^®^T-Easy::Rv2577 was selected and restricted by *Nde*I/*Eco*RV restriction enzymes. The resulting fragment of ∼1.6 kb of the *Rv2577* gene was subcloned in the same *Nde*I/*Eco*RV site into the pML2031 plasmid. The recombinant pML2031::Rv2577 vector with the correct sequence was selected and used to electroporate *M. smegmatis* mc^2^155.

The Rv2577 protein was expressed in the homologous host *M. smegmatis* from the pML2031::Rv2577, which allows its expression as a recombinant protein containing hemagglutinin (HA) and histidine (His) tags (rRv2577). *M. smegmatis* (pML2031::Rv2577) was grown at 37°C until an OD_600 *nm*_ = 1. The expression was induced by the addition of isovalernitrile (IVN) 500 μM and by culturing the bacteria overnight (ON) at 16°C and 150 rpm.

The cell wall fraction (CWF) as well as the soluble cytoplasm and membrane proteins fractions (SF) were obtained as previously described (Forrellad et al., 2014) with some modification. The enrichment in membrane associated proteins was improved by suspending the cells in lysis buffer containing CHAPS (Tris-Cl 50 mM pH 8, NaCl 150 mM, Glycerol 10%, CHAPS 20 mM) as detergent. Then, the cells were treated with lysozyme (1 mg/ml) for 30 min at 4°C, mechanically lysed by Precellys homogenizer and then treated with DNasa (2 μg/ml) for 15 min at 4°C. Subsequently, the cell extract was centrifuged at 6,000 rpm for 20 min at 4°C and the supernatant was collected and centrifuged for a second time at 13,000 rpm for 1 h at 4°C. The supernatant contains the proteins from the SF, whereas the pellet contains the proteins from the CWF. The proteins were separated by 12% SDS-PAGE and Coomassie brilliant blue stained. Detection of rRv2577 was evaluated by Western blotting using the monoclonal α-HA (Sigma) as primary antibody and the α-IgG-mouse conjugated to Alkaline phosphatase (Sigma) as secondary antibody. The antigen-antibody complex was revealed by BCIP/NBT (Promega) reaction performed in alkaline phosphatase buffer.

The molecular weight of Rv2577 was determined performing an Rf vs. Log MW curve, by using the unstained LMW-SDS marker (GE Healthcare) from a 12% SDS-PAGE followed by Coomassie brilliant blue staining. The prestained Blue Plus^®^ Protein Marker (TransGen Biotech) was also included in the gel.

The protein rRv2577 from the soluble fraction was purified by affinity chromatography using an IMAC-Ni^+2^-agarose resin (Qiagen) previously equilibrated in lysis buffer. The recombinant HA-His-tagged protein was eluted whit the same buffer containing 250–500 mM imidazole and then dialyzed against lysis buffer without CHAPs and imidazole. Protein concentration was calculated by Bradford reagent (Bradford, 1976). The SF and CWF obtained from *M. smegmatis* wild type and the recombinant *M. smegmatis* carrying the empty pML2031 vector were used as negative controls as a measured of basal phosphatase and phosphodiesterase activities in mycobacteria.

### Phosphatase Activity

The phosphatase and phosphodiesterase activities were assayed at 37°C in an activity buffer (100 mM Tris-HCl 8 pH, 2 mM MgCl_2_) using the artificial *p*-nitrophenyl phosphate (*p*-NPP) (Sigma-Aldrich SRE0026) or bis-*p*-nitrophenyl phosphate [bis-(*p*-NPP)] (Sigma-Aldrich N3002), respectively, and at a final volume of 200 μl. The enzymatic activity in SF and in the purified rRv2577 was evaluated by adding 100 μg and 1 μgr of the protein, respectively. After 60 min of incubation, the dephosphorylation was monitored by recording the increase of absorbance at 405 nm of *p*-NP. The activity was expressed as ηmol *p*-NP^∗^min^–1*^mg of protein^–1^ using calibrate curves performed with 0, 2, 4, 6, 8, 12, 16 and 20 nmol of *p-*NP in buffers 100 mM MES 6 pH, 100 mM HEPES 7.5 pH and 100 mM Tris-ClH 8 and 9 pH. The same set of buffers was used to determine the optimum pH of rRv2577 activity against the *p*-NPP and bis-(*p*-NPP).

Kinetic parameters were determined under v0 conditions and at a range of substrate concentrations of 0.1–10 mM of *p*-NPP and bis-(*p*-NPP) was used. The kinetic constants K_m_ and V_max_ were calculated using the best fitting curve to the Michaelis-Menten equation by Non-linear regression – Michaelis-Menten in GraphPad Prim 7.0. The k_cat_ was calculated with the equation k_cat_ = V_max_/E_*o*_, with V_max_ values as ηM *p-*NP min^–1^ and Eo as ηM of rRv2577. Control reactions without substrates or the enzyme were included to measure the background level of phosphate. All assays were performed at least in triplicate.

### Nano-HPLC-MS/MS Analysis

The presence of Rv2577 in the SF, CWF and supernatant culture was determined by nano-HPLC-MS/MS. The protein fractions containing 40 μg of proteins were separated by 12% SDS-PAGE followed by staining with Coomassie brilliant blue and Western blotting using the α-HA antibody. Proteins from the supernatant were precipitated by 10% TCA (trichloroacetic acid) treatment at 4°C for O/N, centrifuged at 13,000 rpm and the pellet suspended in Laemmli SDS sample buffer before performing SDS-PAGE. The line from the gel corresponding to each sample of protein was cut in three segments; Cys residues were reduced and alkylated with iodoacetamide and then a gel digestion with trypsin was performed as described previously (Gil et al., 2019). Tryptic peptides were separated using a nano-HPLC system (UltiMate 3000, Thermo Scientific) fitted with a reverse phase nano-column (PepMap RSLC C18, 50 cm × 75 μm ID, 2 μm, Thermo Scientific) and coupled online with a hybrid quadrupole-Orbitrap mass spectrometer (Q-Exactive Plus, Thermo). A linear gradient of solvent B [acetonitrile, 0.1% formic acid (v/v)] from 5 to 55% in 120 min was used. The mass spectrometer was operated in data-dependent acquisition mode. MS/MS of top 12 signals were acquired using a dynamic exclusion list.

A target decoy database was constructed with sequences from *M. smegmatis* strain ATCC 700084/MC^2^155 downloaded from Uniprot and containing Rv2577 sequence and the 127 most common proteomic contaminants. Patternlab for proteomics platform was used from protein identification by database search (FDR < 1% at protein level) (Carvalho et al., 2016). Trypsin was set as the proteolytic enzyme; cysteine carbamidomethylation and methionine oxidation were set as fixed and variable modifications, respectively. Additional searches were performed using the following post-translational modifications: pupylation (K); phosphorylation (S,T,Y); and glycosylation on S, T, and N (DeoxyHex; Hept; Pent; Hex; HexNac; HexN; MurNGlyc; MurNac).

The mass spectrometry proteomics data have been deposited to the ProteomeXchange Consortium via the PRIDE (Perez-Riverol et al., 2019) partner repository with the dataset identifier PXD019769.

### *In silico* Analysis, Structural Characterization, and Site-Directed Mutations

*In silico* analyses of Rv2577 primary sequences were performed using the UniprotKB/Swiss-prot database and the protein-protein BLAST algorithm with the parameter values by default. Conserved Domain Database (CDD) and Pfam in the NCBI and PROSITE in ExPASy were used to identify the conserved domains in Rv2577.

The molecular structure model of Rv2577 was developed with the i-tasser server based on multiple crystallographic structures of related phosphatases. This analysis yielded the PAP from *I. batatas* (protein data bank code; PDB: 1XZW) as the most similar structural analog. The ConSurf server was used to estimate the evolutionary conservation of amino acid residues, by aligning 150 analog proteins of alkaline and acid phosphatases from prokaryotic and eukaryotic organisms, respectively.

The functional and ligand analyses were performed with the COACH predictor [within i-tasser (Yang and Zhang, 2015), by comparisons with the interaction complexes in PDB]. The typical consensus catalytic motif of PAPs and PDEs (GDXX/GDXXY/GNH [D/E]/XXXH/GHXH) is conserved in Rv2577 in the residues D_173_/D_217_/Y_220_/N_253_/H_348_/H_385_ and H_387_. The Site-Directed Mutagenesis Instruction Manual (Stratagene) was used as a guide in order to site-directed mutation methodology to replace the codons for D_173_/D_217_/Y_220_/N_253_/H_348_/H_385_ and H_387_ catalytic residues to those that codify for alanine (Ala).

The vector pML2031::Rv2577 was used as a template and specific primers carrying the mutant codons were designed (Supplementary Table 2). The methylated parental DNA, which carries the wild type sequence of Rv2577, was digested with the enzyme *Dpn*I (Promega) and the newly synthesized mutant vectors were electroporated into *M. smegmatis*. Rv2577 wild type proteins and site-directed mutants were overexpressed and purified as HA-His fusion proteins. The phosphatase and phosphodiesterase activities of the recombinant proteins were evaluated with *p*-NPP and bis-(*p*-NPP) substrates, respectively, as described above.

### Rv2577 Gene Expression, RNA Extraction and RT-PCR

The *Rv2577* gene expression was determined *in vitro* during the growth culture of *Mtb* CDC 1551 strain in rich 7H9-AD-T-G media. The bacterium was cultured to exponential phase of ÕD_600 nm_0.3–0.5. Reached this point, total RNA was obtained as described by Santangelo et al. (2002).

*In vivo* expression of *Rv2577* was determined during the intracellular growth of *Mtb* CDC 1551 strain after 5 h of infection of human THP-1 macrophages. Total RNA was obtained as previously described (Blanco et al., 2009). cDNA was obtained using 1 μg (*in vitro*) or 300 ηg (*in vivo*) of total RNA and reverse transcribed using the M-MLV reverse transcriptase (Promega). The *Rv2577* expression was determined by end point PCR (RT-PCR) using the Rv2577 F (5′ATTGCGATCGAGCGTATTGC3′) and Rv2577 R (5′AATTCGGTCTTGTGCCAGGT3′) pair of primers as described by Blanco et al. (2009). The expression of the housekeeping *sigA* gene was used as control of transcription.

### Cellular Traffic, Indirect Immunofluorescence, and Confocal Microscopy

The *Mtb* CDC 1551 wild type, Rv2577 mutant and complemented strains (Supplementary Table 2) were covalently stained with fluorescein isothiocyanate (FITC) (Sigma-Aldrich, 3326-32-7) as previously described (Forrellad et al., 2013a). The stained bacteria were used to infect human THP-1 or murine J774 macrophages. Before infections, THP-1 cells were activated by phorbol 12-myristate 13-acetate (PMA) treatment, 20 nM for 48 h. Both macrophage cell lines were infected at a MOI of 5:1 (bacterium:macrophage) and incubated in RPMI medium for 1 h at 37°C under a 5% CO_2_ atmosphere (uptake). The infected macrophages were washed to eliminate the extracellular bacteria and incubated for 2 h (chase). Indirect immunofluorescence was performed on the infected cells as previously described (Forrellad et al., 2013a). THP-1 macrophages were marked using anti-LAMP-3 (Santa Cruz Biotechnology, Inc.) (1:50 diluted in PBS), as primary antibody, and anti-goat Cy5-conjugated (1:500 diluted in PBS), as secondary antibody (Jackson Immuno Research Inc.). On the other hand, J774 macrophages were marked using anti-LAMP-2, as primary antibody, (the Developmental Studies Hybridoma Bank, developed under the auspices of the NICHD and maintained by The University of Iowa, Department of Biology, Iowa City, IA 52242) and anti-rat Cy5-conjugated (Jackson Immuno Research Inc.), as secondary antibody.

The cells were analyzed by confocal microscopy using a Leica TCS-SP5 spectral confocal microscope (Leica Microsystems) at the integrated Microscopy Laboratory (INTA Castelar, Argentina). Mycobacterial internalization was analyzed and quantified using Fiji software (U.S. National Institutes of Health, Bethesda, MD) as described previously (Forrellad et al., 2013a). The experiments were performed in duplicates and three independent infections were performed for each assay.

The statistical analysis was performed using analysis of variance (ANOVA) and Bonferroni’s post-tests. Fluorescence intensity values were plotted and analyzed using GraphPad Prism 7 (GraphPad Software, Inc.).

### Macrophage Infection

Human THP-1, stimulated by PMA treatment (as described above), and murine J774 macrophages were infected with either *Mtb* CDC 1551 wild type, Rv2577 mutant and complemented strains (Supplementary Table 2) at a MOI of 0.1:1 and incubated in RPMI medium for 1 h at 37°C under a 5% CO_2_ atmosphere (uptake). The infected macrophages were washed with PBS 1X and incubated for 2 h (chase) in RPMI media supplemented with 10 μg/ml gentamicin to eliminate the extracellular bacteria. At 3, 48, 96, and 120 h post-infection (hpi) the bacteria were collected, serial diluted and plated in 7H10-AD-G-T medium. Colony formatting units (CFUs) were assessed after 30 days of incubation at 37°C.

### Mouse Infection

BALB/c mice (6–8 weeks old, *n* = 6) were intratracheally infected with *Mtb* CDC 1551 (wild type), Rv2577 mutant or the complemented strains (Supplementary Table 2). For this purpose, the bacteria were disaggregated and resuspended in PBS buffer. The selected infective doses were 1^∗^10^3^ bacteria/100 μl of buffer per mouse. At 1 and 30 days post-infection (dpi), the mice were sacrificed and the lungs and spleen were extracted, homogenized and plated on 7H10-G-T-ADC solid medium to determine the colony forming units (CFUs).

The experiments were performed in compliance with the regulations of the Institutional Animal Care and Use Committee (CICUAE) of INTA.

## Data Availability Statement

The original contributions presented in the study are publicly available. This data can be found here: https://www.ebi.ac.uk/pride/.

## Ethics Statement

The animal study was reviewed and approved by Institutional Animal Care and Use Committee (CICUAE) of INTA.

## Author Contributions

MAF, FCB, CLV, AY and FB were CONICET fellows. MAF performed the experiments. FCB and EAG collaborated in the mice infection. RMDdV made the 3D model. CLV and MGG participated in the images design of confocal microscopy. RD performed the nano-HPLC-MS/MS analysis. AY collaborated in the biochemistry studies. MAF, AV and FB designed and drafted the manuscript. All authors contributed to the article and approved the submitted version.

## Conflict of Interest

The authors declare that the research was conducted in the absence of any commercial or financial relationships that could be construed as a potential conflict of interest.

## Footnotes

1 https://www.who.int/tb/publications/global_report/en/

2 https://mycobrowser.epfl.ch/

3 https://www.ncbi.nlm.nih.gov/Structure/cdd/cddsrv.cgi

## Supplementary Material

Supplementary Figure 1 Blastp alignment of Rv2577 protein. The sequence of *M. tuberculosis* Rv2577 protein was used as query to perform a protein-protein BLAST alignment by choosing UniprotKB/Swiss-prot database. The extent of the alignment on the Rv2577 sequence is represented by color lines. The alignment score between 80–200 (pink) and 50–80 (green) represented Purple acid phosphatases (PAP) from eukaryote organisms: *Glicyne max*, *Phaseolus vulgaris*, *Ipomoea batatas*, *Arabidopsis thaliana*, *Allium cepa*, and *Dario rerio*. The proteins have an identity range from 28.88 to 22.39%. The conserved Purple acid phosphatases N-terminal and MPP_superfamily of metallophosphatases or Calcineurin-like phosphoesterase domains are located in residues 67–150 and 215–513 from Rv2577 sequence, respectively. Proteins with the worst extent alignment score < 40 (black lines) are represented by a Probable lactoylglutathione lyase and thymidylate synthase.

Supplementary Figure 2 rRv2577 overexpressed in *M. smegmatis* occurs as two isoforms. 12% SDS-PAGE of soluble (SF) (inner membrane and cytosolic) and cell wall proteins (CWF) from *M. smegmatis* wild type (Ms), *M. smegmatis* carrying the empty pML2031 vector (2031) and *M. smegmatis* carrying the recombinant pML2031:Rv2577 vector (Rv2577) were transferred to a nitrocellulose membrane and stained by Ponceau red staining (left panel). The same protein amount (100 μg) was loaded per lane. The membrane was submitted to Western blot using α-HA as primary antibody/α-IgG mouse^∗^alkaline phosphatase conjugated as secondary antibody, and reveled with BCIP/NBT substrate (right panel). The arrows show the rRv2577 isoform of 60.2 kDa from the soluble fraction (SF. Rv2577) and the alternative 64.5 kDa protein from cell wall (CWF. Rv2577). MW. Low Molecular Weight (LMW) SDS marker (GE Healthcare).

Supplementary Figure 3 The rRv2577 activities are favored at alkaline pHs. Phosphatase and phosphodiesterase activities of purified rRv2577 protein were monitored at 6, 7.5, 8, and 9 pHs using *p*-NPP (circle) and bis-(*p*-NPP) (square) substrates, respectively. rRv2577 presents a higher activity at physiological and alkaline pH for both analyzed substrates.

Supplementary Figure 4 Kinetic characterization of rRv2577. **(A)** Reaction rates of rRv2577 using *p-*NPP (15 mM) as substrate showing the linear behavior throughout the reaction time. In addition, the graph displays the enzyme activity increase (slope of each curve) as a function of enzyme concentration present in the assay, tested for 0 (circles), 0.5 (squares) and 1 μg (triangles) expressed as ηmol of *p*-NP. **(B)** Michaelis-Menten kinetic representation of purified rRv2577 using *p-*NPP (circles) and bis-(*p-*NPP) (triangles) as substrates. The kinetic constants K_m_ and V_max_ were calculated using the best fitting curve to the Michaelis-Menten equation (Non-linear regression – Michaelis-Menten in GraphPad Prim 7.0). Reaction rates of rRv2577 were expressed as specific activity (ηmoles *p-*NP^∗^min^–1*^mg^–1^ of protein). The k_cat_ was calculated with the equation k_cat_ = V_max_/E_*o*_, with V_max_ values as ηM *p-*NP s^–1^ and Eo as ηM of rRv2577.

Supplementary Figure 5 Expression of Rv2577 wild type and site-directed mutants in *M. smegmatis*. Western blot analysis with α-HA antibody (top panel) of the soluble (inner membrane and cytoplasm) **(A)** and cell wall **(B)** fractions of proteins from *M. smegmatis* (Ms) and *M. smegmatis* overexpressing the Rv2577 wild type (Rv2577wt) or its site-directed mutants D_173_A, D_217_A, N_253_A, H_385_A, Y_220_A, H_348_A, and H_387_A. The arrows indicate the presence of Rv2577. The same protein amount (100 μg) was loaded per lane, as shown by Ponceau red staining (bottom panel in **A**) and **(B)**. Mw: Prestained Blue plus^®^ protein marker 14–100 kDa (TransGen Biotech) **(A)** or unstained LMW-SDS marker (GE Healthcare) **(B)**.

Supplementary Figure 6 The integrity of the Rv2577 gene in the *M. tuberculosis* strains. **(A)** PCR amplification of the kanamycin resistance (Km^*R*^) from the genomes of *M. tuberculosis* CDC 1551 wild type (Mtb), Rv2577 mutant (Rv2577-mut) and complemented (Rv2577-comp) strains. A4̃00 bp fragment of the Km^*R*^ was amplified from the Rv2577 mutant and complemented strains (arrow). **(B)** PCR amplification of *Rv2577* from the *M. tuberculosis* strains studied in A); a 1590 bp fragment corresponding to the full *Rv2577* gene was amplified from the *M. tuberculosis* wild type and complemented strains (arrow). Mw: DNA marker 1 kb (Roche). C+ : plasmid pML2031:Rv2577 was used as a positive control.

Supplementary Figure 7 Growth kinetics *of* the *M. tuberculosis* strains. *Mtb* CDC 1551 (*Mtb*, black), Rv2577 mutant (Rv2577-mut, blue) and complemented (Rv2577-comp, orange) strains were growth in 7H9 rich media as described in section “Materials and Methods.” The bacterial growth kinetic was monitored by OD_600 *nm*_ measurement at different times (days). The data are representative of three independent experiments performed in duplicate.

Supplementary Figure 8 *Rv2577* gene is transcribed during the *in vitro* and *in vivo* growth of *Mtb*. **(A)** RT-PCR of Rv2577 gene in a 2% agarose gel electrophoresis. Total RNA and cDNA were obtained from *Mtb* CDC 1551 bacterium growing *in vitro* into rich media (*in vitro*) or during its intracellular growth after 5 h post-infection in human THP-1 macrophages (*in vivo*). The 81 bp fragment corresponding to the *Rv2577* gene is shown by the arrow (Rv2577). The pML2031:Rv2577 vector (C+ p) and the genomic DNA from *Mtb* CDC 1551 strain (C+) were used as positive controls. Water was used in the PCR mix as a negative control (C−). **(B)** The 110 bp PCR fragment from the housekeeping *sigA* gene (*sigA, arrow*) was used as a control of transcription. Mw: 100 bp DNA ladder (TransGene Biotech).

Supplementary Figure 9 Rv2577 participates in the arrest of murine phagosome maturation. **(A)** Mouse J774 macrophages were infected with *M. tuberculosis* wild type (Mtb) as well as the Rv2577 mutant (Rv2577-mut) and the complemented (Rv2577-comp) strains to analyze the intracellular trafficking. The bacteria were labeled with FITC (green) and LAMP-2 protein was detected using a specific antibody (red). **(B)** Quantification of the fluorescence observed in **(A)** the association of LAMP-2 to the mycobacterial compartment. The data are based on a representative experiment of three independent experiments, all performed in duplicate. ^∗∗∗^*p* < 0.001 statistical analysis using ANOVA. Number of counted phagosomes = 150 from at least 50 different macrophages.

Supplementary Figure 10 *Ex vivo* survival of *M. tuberculosis* strains in human THP-1 macrophages. Survival of *M. tuberculosis* CDC 1551 wild type (Mtb), Rv2577 mutant (Rv2577-mut) and complemented (Rv2577-comp) strains measured as CFU^∗^ml^–1^ in human THP-1 macrophages after 3, 48, 96, and 120 h post-infection (hpi). Data show a representative of three experiments performed independently in duplicate. No significant differences were observed by ANOVA analysis.

Supplementary Table 1 Nano-HPLC-MS.MS analysis of Rv2577. Proteins identified in soluble (line 3 to 126) and cell wall fraction (line 129 to 691) from *M. smegmatis* expressing Rv2577. Proteins identified in supernatants from *M.smegmatis* expressing Rv2577.

Supplementary Table 2 Strains, plasmids and primers used in this study.

## References

[B1] Arora G. Tiwari P. Mandal R. S. Gupta A. Sharma D. Saha S. (2014). High throughput screen identifies small molecule inhibitors specific for *Mycobacterium tuberculosis* phosphoserine phosphatase. *J. Biol.Chem*. 289 25149–25165. 10.1074/jbc.M114.597682 25037224 PMC4155679

[B2] Ashkenazy H. Abadi S. Martz E. Chay O. Mayrose I. Pupko T. (2016). ConSurf 2016: an improved methodology to estimate and visualize evolutionary conservation in macromolecules. *Nucleic Acids Res.* 44 W344–W350. 10.1093/nar/gkw408 27166375 PMC4987940

[B3] Bach H. Papavinasasundaram K. G. Wong D. Hmama Z. Av-Gay Y. (2008). *Mycobacterium tuberculosis* virulence is mediated by PtpA dephosphorylation of human vacuolar protein sorting 33B. *Cell Host Microbe* 3 316–322. 10.1016/j.chom.2008.03.008 18474358

[B4] Beresford N. Patel S. Armstrong J. Szöor B. Fordham-Skelton A. P. Tabernero L. (2007). MptpB, a virulence factor from *Mycobacterium tuberculosis*, exhibits triple-specificity phosphatase activity. *Biochem. J.* 406 13–18. 10.1042/BJ20070670 17584180 PMC1948985

[B5] Beresford N. J. Mulhearn D. Szczepankiewicz B. Liu G. Johnson M. E. Fordham-Skelton A. (2009). Inhibition of MptpB phosphatase from *Mycobacterium tuberculosis* impairs mycobacterial survival in macrophages. *J. Antimicrob. Chemother.* 63 928–936. 10.1093/jac/dkp031 19240079

[B6] Birhanu A. G. Yimer S. A. Kalayou S. Riaz T. Zegeye E. D. Holm-Hansen C. (2019). Ample glycosylation in membrane and cell envelope proteins may explain the phenotypic diversity and virulence in the *Mycobacterium tuberculosis* complex. *Sci. Rep.* 9 1–15. 10.1038/s41598-019-39654-9 30814666 PMC6393673

[B7] Blanco F. C. Nunez-García J. García-Pelayo C. Soria M. Bianco M. V. Zumárraga M. (2009). Differential transcriptome profiles of attenuated and hypervirulent strains of *Mycobacterium bovis*. *Microbes Infect.* 11 956–963. 10.1016/j.micinf.2009.06.006 19591956

[B8] Boitel B. Ortiz-Lombardía M. Durán R. Pompeo F. Cole S. T. Cerveñansky C. (2003). PknB kinase activity is regulated by phosphorylation in two thr residues and dephosphorylation by PstP, the cognate phospho-ser/Thr phosphatase, in *Mycobacterium tuberculosis*. *Mol. Microbiol.* 49 1493–1508. 10.1046/j.1365-2958.2003.03657.x 12950916

[B9] Bradford M. (1976). A rapid and sensitive method for the quantitation of microgram quantities of protein utilizing the principle of protein-dye binding. *Anal. Biochem.* 72 248–254. 10.1006/abio.1976.9999 942051

[B10] Carvalho P. C. Lima D. B. Leprevost F. V. Santos M. D. M. Fischer J. S. G. Aquino P. F. (2016). Integrated analysis of shotgun proteomic data with patternlab for proteomics 4.0. *Nat. Protoc.* 11 102–117. 10.1038/nprot.2015.133 26658470 PMC5722229

[B11] Chakraborti P. K. (2016). Eukaryotic-type ser/thr protein kinase mediated phosphorylation of mycobacterial phosphodiesterase affects its localization to the cell. *Front. Microbiol.* 7:123. 10.3389/fmicb.2016.00123 26904001 PMC4746578

[B12] Cole S. T. Brosch R. Parkhill J. Garnier T. Churcher C. Harris D. (1998). Deciphering the biology of *Mycobacterium tuberculosis* from the complete genome sequence [See Comments] [Published Erratum Appears in Nature 1998 Nov 12. *Nature* 396 537–544. 10.1038/31159 9634230

[B13] Dey R. J. Dey B. Zheng Y. Cheung L. S. Zhou J. Sayre D. (2017). Inhibition of innate immune cytosolic surveillance by an *M.tuberculosis* phosphodiesterase. *Nat. Chem. Biol.* 13 210–217. 10.1038/nchembio.2254 28106876

[B14] Diethmaier C. Newman J. A. Kovács ÁT. Kaever V. Herzberg C. Rodrigues C. (2014). The YmdB phosphodiesterase is a global regulator of late adaptive responses in *Bacillus subtilis*. *J. Bacteriol.* 196 265–275. 10.1128/JB.00826-13 24163345 PMC3911264

[B15] Fan L. Wu X. Jin C. Li F. Xiong S. Dong Y. (2018). MptpB promotes mycobacteria survival by inhibiting the expression of inflammatory mediators and cell apoptosis in macrophages. *Front. Cell. Infect. Microbiol.* 8:171. 10.3389/fcimb.2018.00171 29888212 PMC5981270

[B16] Fernandez-Soto P. Bruce A. J. E. Fielding A. J. Cavet J. S. Tabernero L. (2019). Mechanism of catalysis and inhibition of *Mycobacterium tuberculosis* SapM, implications for the development of novel antivirulence drugs. *Sci. Rep.* 9 1–14. 10.1038/s41598-019-46731-6 31312014 PMC6635428

[B17] Flores-Díaz M. Monturiol-Gross L. Naylor C. Alape-Girón A. Flieger A. (2016). Bacterial sphingomyelinases and phospholipases as virulence factors. *Microbiol. Mol. Biol. Rev.* 80 597–628. 10.1128/mmbr.00082-15 27307578 PMC4981679

[B18] Forrellad M. A. Bianco M. V. Blanco F. C. Nuñez J. Klepp L. I. Vazquez C. L. (2013a). Study of the in vivo role of Mce2R, the transcriptional regulator of Mce2 operon in *Mycobacterium tuberculosis*. *BMC Microbiol.* 13:200. 10.1186/1471-2180-13-200 24007602 PMC3847441

[B19] Forrellad M. A. Klepp L. I. Gioffré A. García J. S. Morbidoni H. R. Paz Santangelo M. D. L. (2013b). Virulence factors of the *Mycobacterium tuberculosis* complex. *Virulence* 4 3–66. 10.4161/viru.22329 23076359 PMC3544749

[B20] Forrellad M. A. McNeil M. Santangelo M. D. L. Blanco F. C. García E. Klepp L. I. (2014). Role of the Mce1 transporter in the lipid homeostasis of *Mycobacterium tuberculosis*. *Tuberculosis* 94 170–177. 10.1016/j.tube.2013.12.005 24440549 PMC3951760

[B21] Gil M. Lima A. Rivera B. Rossello J. Urdániz E. Cascioferro A. (2019). New substrates and interactors of the mycobacterial serine/threonine protein kinase pkng identified by a tailored interactomic approach. *J. Proteomics* 192 321–333. 10.1016/j.jprot.2018.09.013 30267874

[B22] Jackson M. (2014). The mycobacterial cell envelope-lipids. *Cold Spring Harb. Perspect. Med.* 4 1–22. 10.1101/cshperspect.a021105 25104772 PMC4200213

[B23] Johnson R. M. McDonough K. A. (2018). Cyclic nucleotide signaling in *Mycobacterium tuberculosis*: an expanding repertoire. *Pathog. Dis.* 76 1–12. 10.1093/femspd/fty048 29905867 PMC6693379

[B24] Keppetipola N. Shuman S. (2008). A phosphate-binding histidine of binuclear metallophosphodiesterase enzymes is a determinant of 2′,3′-cyclic nucleotide phosphodiesterase activity. *J. Biol. Chem.* 283 30942–30949. 10.1074/jbc.M805064200 18757371 PMC2576524

[B25] Malhotra N. Karthikeyan S. Chakraborti P. K. (2017). Phosphorylation of mycobacterial phosphodiesterase by eukaryotic-type Ser/Thr kinase controls its two distinct and mutually exclusive functionalities. *J. Biol. Chem.* 292 17362–17374. 10.1074/jbc.M117.784124 28855253 PMC5655513

[B26] Margenat M. Labandera A. M. Gil M. Carrion F. Purificação M. Razzera G. (2015). New potential eukaryotic substrates of the mycobacterial protein tyrosine phosphatase PtpA: hints of a bacterial modulation of macrophage bioenergetics state. *Sci. Rep.* 5 1–11. 10.1038/srep08819 25743628 PMC5390082

[B27] Perez-Riverol Y. Csordas A. Bai J. Bernal-Llinares M. Hewapathirana S. Kundu D. J. (2019). The PRIDE database and related tools and resources in 2019: improving support for quantification data. *Nucleic Acids Res.* 47 D442–D450. 10.1093/nar/gky1106 30395289 PMC6323896

[B28] Puri R. V. Reddy P. V. Tyagi A. K. (2013). Secreted acid phosphatase (SapM) of *Mycobacterium tuberculosis* is indispensable for arresting phagosomal maturation and growth of the pathogen in guinea pig tissues. *PLoS One* 8:e0070514. 10.1371/journal.pone.0070514 23923000 PMC3724783

[B29] Richter W. (2002). 3′,5′-cyclic nucleotide phosphodiesterases class III: members, structure, and catalytic mechanism. *Proteins Struct. Funct. Genet.* 46 278–286. 10.1002/prot.10049 11835503

[B30] Rodriguez F. Lillington J. Johnson S. Timmel C. R. Lea S. M. Berks B. C. (2014). Crystal structure of the *Bacillus subtilis* phosphodiesterase PhoD reveals an iron and calcium-containing active site. *J. Biol. Chem.* 289 30889–30899. 10.1074/jbc.M114.604892 25217636 PMC4223295

[B31] Saleh M. T. Belisle J. T. (2000). Secretion of an acid phosphatase (SapM) by *Mycobacterium tuberculosis* that is similar to eukaryotic acid phosphatases. *J. Bacteriol.* 182 6850–6853. 10.1128/JB.182.23.6850-6853.2000 11073936 PMC111434

[B32] Santangelo M. P. Goldstein J. Alito A. Gioffre A. Romano M. I. Caimi K. (2002). Negative transcriptional regulation of the Mce3 operon in *Mycobacterium tuberculosis*. *Microbiology* 148 2997–3006. 10.1099/00221287-148-10-2997 12368433

[B33] Schenk G. Boutchard C. L. Carrington L. E. Noble C. J. Moubaraki B. Murray K. S. (2001). A purple acid phosphatase from sweet potato contains an antiferromagnetically coupled binuclear fe-mn center. *J. Biol. Chem.* 276 19084–19088. 10.1074/jbc.M009778200 11278566

[B34] Schenk G. Gahan L. R. Carrington L. E. Mitič N. Valizadeh M. Hamilton S. E. (2005). Phosphate forms an unusual tripodal complex with the Fe-Mn center of sweet potato purple acid phosphatase. *Proc. Natl. Acad. Sci. U.S.A.* 102 273–278. 10.1073/pnas.0407239102 15625111 PMC544300

[B35] Shenoy A. R. Capuder M. Draškovič P. Lamba D. Visweswariah S. S. Podobnik M. (2007). Structural and biochemical analysis of the Rv0805 cyclic nucleotide phosphodiesterase from *Mycobacterium tuberculosis*. *J. Mol. Biol.* 365 211–225. 10.1016/j.jmb.2006.10.005 17059828

[B36] Shenoy A. R. Sreenath N. Podobnik M. Kovačevič M. Visweswariah S. S. (2005). The Rv0805 gene from *Mycobacterium tuberculosis* encodes a 3′,5′-cyclic nucleotide phosphodiesterase: biochemical and mutational analysis. *Biochemistry* 44 15695–15704. 10.1021/bi0512391 16313172

[B37] Singh R. Rao V. Shakila H. Gupta R. Khera A. Dhar N. (2003). Disruption of MptpB impairs the ability of *Mycobacterium tuberculosis* to survive in guinea pigs. *Mol. Microbiol.* 50 751–762. 10.1046/j.1365-2958.2003.03712.x 14617138

[B38] Traini M. Kumaran R. Thaysen-Andersen M. Kockx M. Jessup W. Kritharides L. (2017). N-glycosylation of human sphingomyelin phosphodiesterase acid-like 3A (SMPDL3A) is essential for stability, secretion and activity. *Biochem. J.* 474 1071–1092. 10.1042/BCJ20160735 28104755

[B39] Vergne I. Chua J. Lee H. H. Lucas M. Belisle J. Deretic V. (2005). Mechanism of phagolysosome biogenesis block by viable *Mycobacterium tuberculosis*. *Proc. Natl. Acad. Sci. U.S.A.* 102 4033–4038. 10.1073/pnas.0409716102 15753315 PMC554822

[B40] Wang J. Ge P. Qiang L. Tian F. Zhao D. Chai Q. (2017). The mycobacterial phosphatase PtpA regulates the expression of host genes and promotes cell proliferation. *Nat. Commun.* 8:244. 10.1038/s41467-017-00279-z 28811474 PMC5557760

[B41] Wang L. K. Smith P. Shuman S. (2013). Structure and mechanism of the 2′,3′ phosphatase component of the bacterial Pnkp-Hen1 RNA repair system. *Nucleic Acids Res.* 41 5864–5873. 10.1093/nar/gkt221 23595150 PMC3675462

[B42] Wehenkel A. Bellinzoni M. Schaeffer F. Villarino A. Alzari P. M. (2007). Structural and binding studies of the three-metal center in two mycobacterial PPM Ser/Thr protein phosphatases. *J. Mol. Biol.* 374 890–898. 10.1016/j.jmb.2007.09.076 17961594

[B43] Williams R. S. Moncalian G. Williams J. S. Yamada Y. Limbo O. Shin D. S. (2008). Mre11 dimers coordinate DNA end bridging and nuclease processing in double-strand-break repair. *Cell* 135 97–109. 10.1016/j.cell.2008.08.017 18854158 PMC2681233

[B44] Wong D. Bach H. Sun J. Hmama Z. Av-Gay Y. (2011). *Mycobacterium tuberculosis* protein tyrosine phosphatase (PtpA) excludes host vacuolar-H +-ATPase to inhibit phagosome acidification. *Proc. Natl. Acad. Sci. U.S.A.* 108 19371–19376. 10.1073/pnas.1109201108 22087003 PMC3228452

[B45] Wong D. Chao J. D. Av-Gay Y. (2013). *Mycobacterium tuberculosis*-secreted phosphatases: from pathogenesis to targets for TB drug development. *Trends Microbiol.* 21 100–109. 10.1016/j.tim.2012.09.002 23084287

[B46] Yang J. Zhang Y. (2015). I-TASSER server: new development for protein structure and function predictions. *Nucleic Acids Res.* 43 W174–W181. 10.1093/nar/gkv342 25883148 PMC4489253

[B47] Yeung S. L. Cheng C. Lui T. K. O. Tsang J. S. H. Chan W. T. Lim B. L. (2009). Purple acid phosphatase-like sequences in prokaryotic genomes and the characterization of an atypical purple alkaline phosphatase from burkholderia cenocepacia J2315. *Gene* 440 1–8. 10.1016/j.gene.2009.04.002 19376213

[B48] Zhang Y. Skolnick J. (2004). Scoring function for automated assessment of protein structure template quality. *Proteins Struct. Funct. Genet.* 57 702–710. 10.1002/prot.20264 15476259

[B49] Zulauf K. E. Sullivan J. T. Braunstein M. (2018). The SecA2 pathway of *Mycobacterium tuberculosis* exports effectors that work in concert to arrest phagosome and autophagosome maturation. *PLoS Pathog.* 14:e1007011. 10.1371/journal.ppat.1007011 29709019 PMC5945054

