# Supplementary figures and images for: Rv2577 of Mycobacterium tuberculosis Is a Virulence Factor With Dual Phosphatase and Phosphodiesterase Functions
Source: Front Microbiol. 2020 Oct 22;11:570794. doi: 10.3389/fmicb.2020.570794 (PMC7642983; doi:10.3389/fmicb.2020.570794)

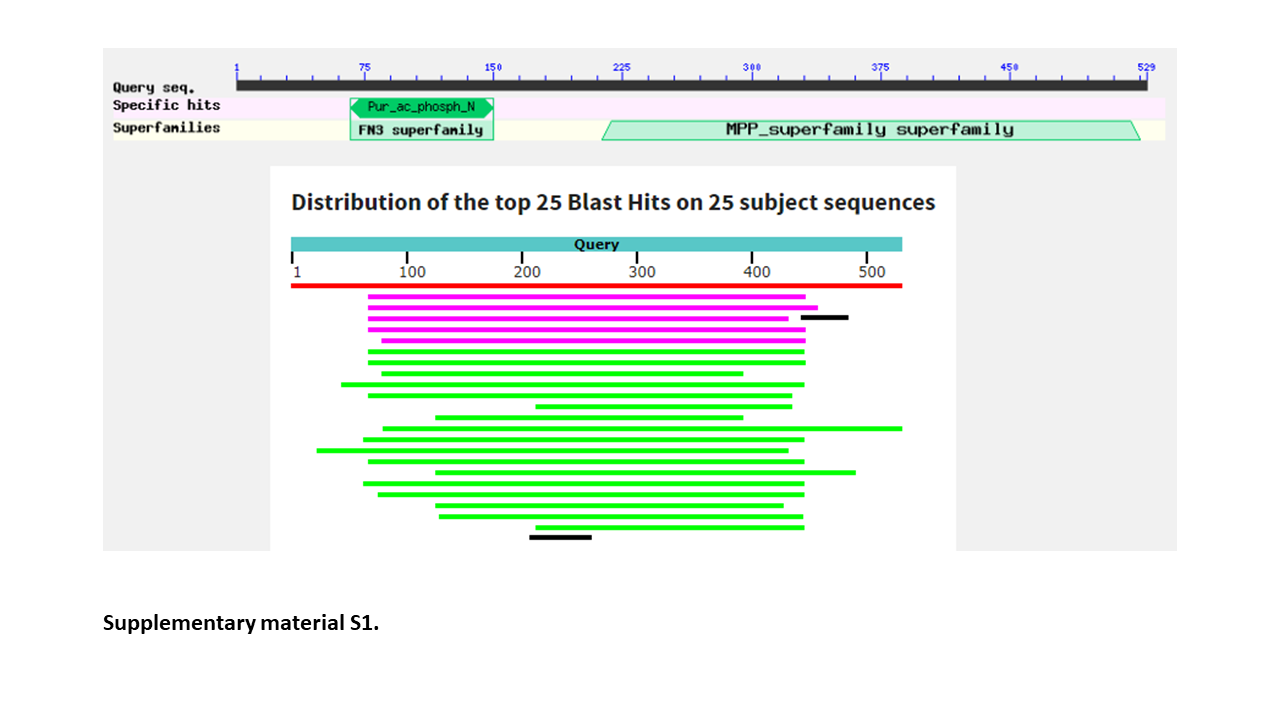

Supplement: Supplementary Figure 1 — Blastp alignment of Rv2577 protein. The sequence of M. tuberculosis Rv2577 protein was used as query to perform a protein-protein BLAST alignment by choosing UniprotKB/Swiss-prot database. The extent of the alignment on the Rv2577 sequence is represented by color lines. The alignment score between 80–200 (pink) and 50–80 (green) represented Purple acid phosphatases (PAP) from eukaryote organisms: Glicyne max, Phaseolus vulgaris, Ipomoea batatas, Arabidopsis thaliana, Allium cepa, and Dario rerio. The proteins have an identity range from 28.88 to 22.39%. The conserved Purple acid phosphatases N-terminal and MPP_superfamily of metallophosphatases or Calcineurin-like phosphoesterase domains are located in residues 67–150 and 215–513 from Rv2577 sequence, respectively. Proteins with the worst extent alignment score < 40 (black lines) are represented by a Probable lactoylglutathione lyase and thymidylate synthase. [file Image_1.TIF]

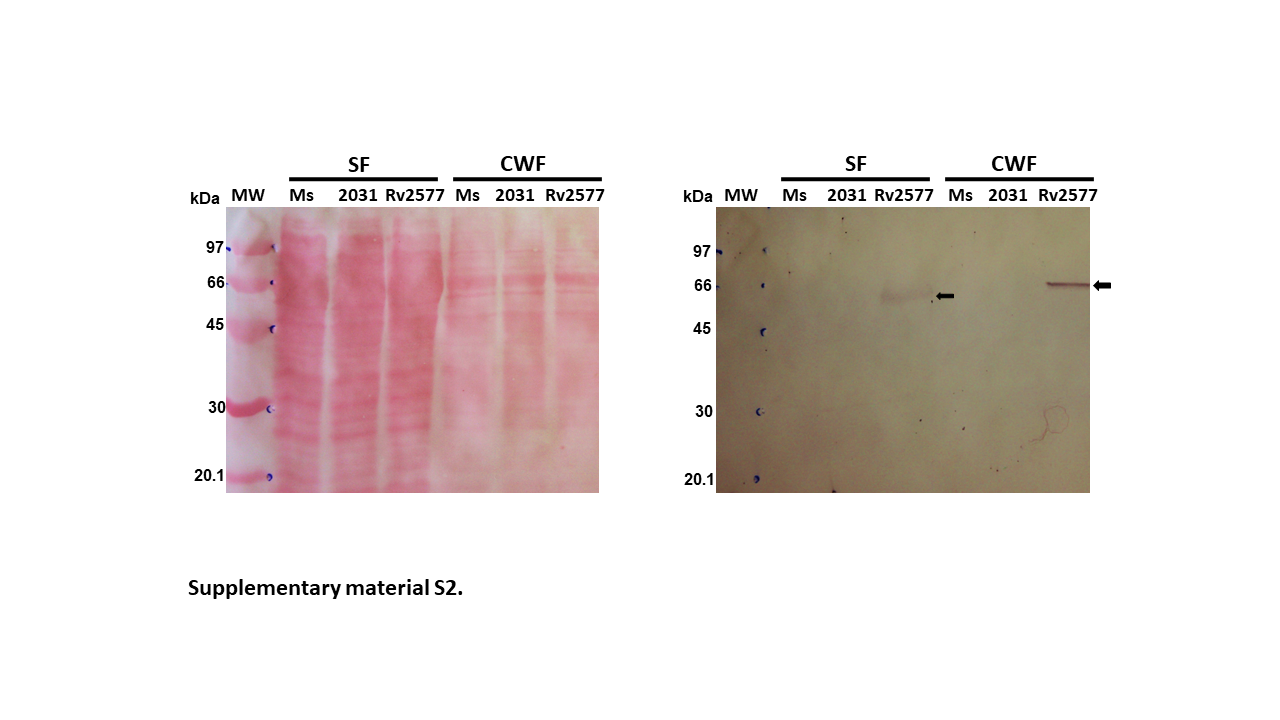

Supplement: Supplementary Figure 2 — rRv2577 overexpressed in M. smegmatis occurs as two isoforms. 12% SDS-PAGE of soluble (SF) (inner membrane and cytosolic) and cell wall proteins (CWF) from M. smegmatis wild type (Ms), M. smegmatis carrying the empty pML2031 vector (2031) and M. smegmatis carrying the recombinant pML2031:Rv2577 vector (Rv2577) were transferred to a nitrocellulose membrane and stained by Ponceau red staining (left panel). The same protein amount (100 μg) was loaded per lane. The membrane was submitted to Western blot using α-HA as primary antibody/α-IgG mouse∗alkaline phosphatase conjugated as secondary antibody, and reveled with BCIP/NBT substrate (right panel). The arrows show the rRv2577 isoform of 60.2 kDa from the soluble fraction (SF. Rv2577) and the alternative 64.5 kDa protein from cell wall (CWF. Rv2577). MW. Low Molecular Weight (LMW) SDS marker (GE Healthcare). [file Image_2.TIF]

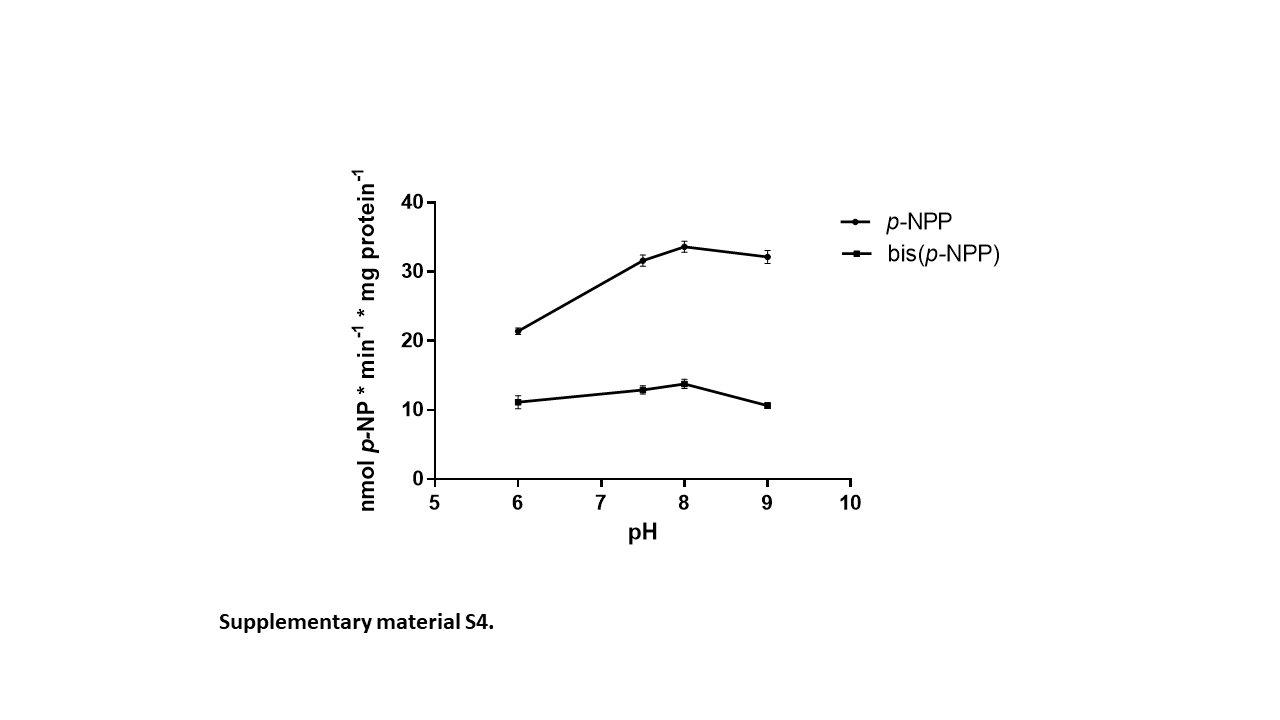

Supplement: Supplementary Figure 3 — The rRv2577 activities are favored at alkaline pHs. Phosphatase and phosphodiesterase activities of purified rRv2577 protein were monitored at 6, 7.5, 8, and 9 pHs using p-NPP (circle) and bis-(p-NPP) (square) substrates, respectively. rRv2577 presents a higher activity at physiological and alkaline pH for both analyzed substrates. [file Image_3.TIF]

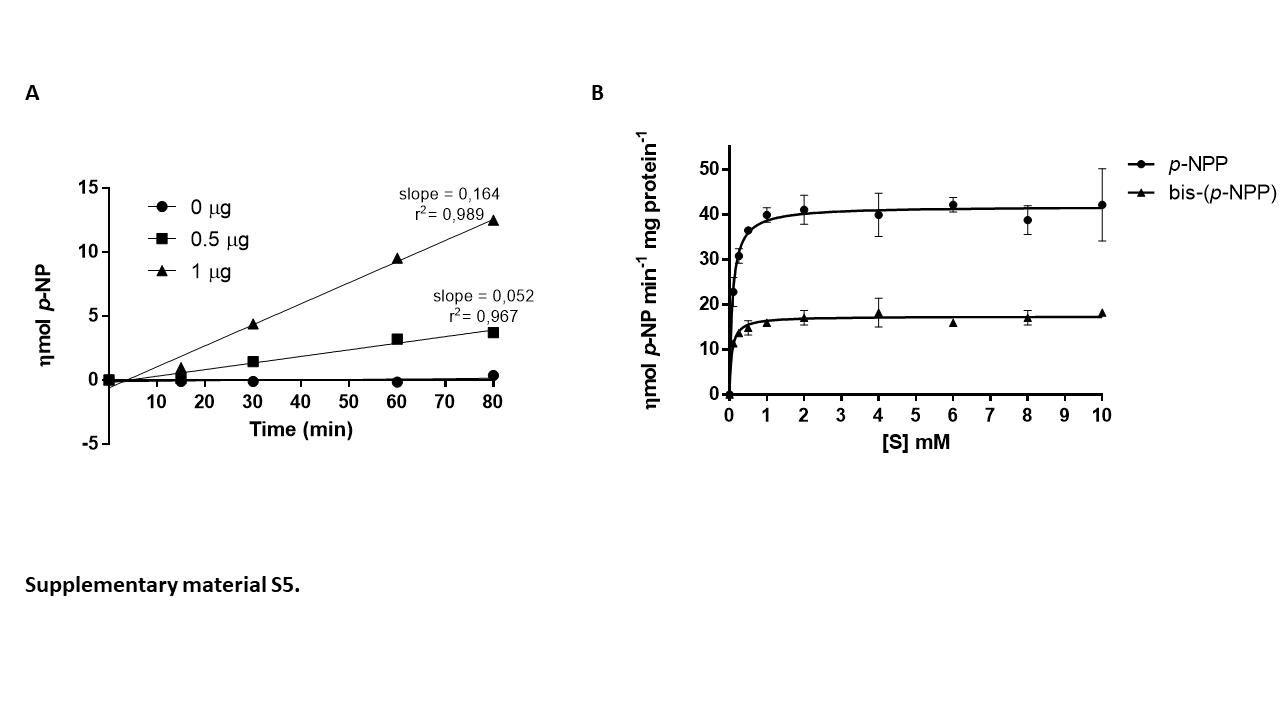

Supplement: Supplementary Figure 4 — Kinetic characterization of rRv2577. (A) Reaction rates of rRv2577 using p-NPP (15 mM) as substrate showing the linear behavior throughout the reaction time. In addition, the graph displays the enzyme activity increase (slope of each curve) as a function of enzyme concentration present in the assay, tested for 0 (circles), 0.5 (squares) and 1 μg (triangles) expressed as ηmol of p-NP. (B) Michaelis-Menten kinetic representation of purified rRv2577 using p-NPP (circles) and bis-(p-NPP) (triangles) as substrates. The kinetic constants Km and Vmax were calculated using the best fitting curve to the Michaelis-Menten equation (Non-linear regression – Michaelis-Menten in GraphPad Prim 7.0). Reaction rates of rRv2577 were expressed as specific activity (ηmoles p-NP∗min–1*mg–1 of protein). The kcat was calculated with the equation kcat = Vmax/Eo, with Vmax values as ηM p-NP s–1 and Eo as ηM of rRv2577. [file Image_4.TIF]

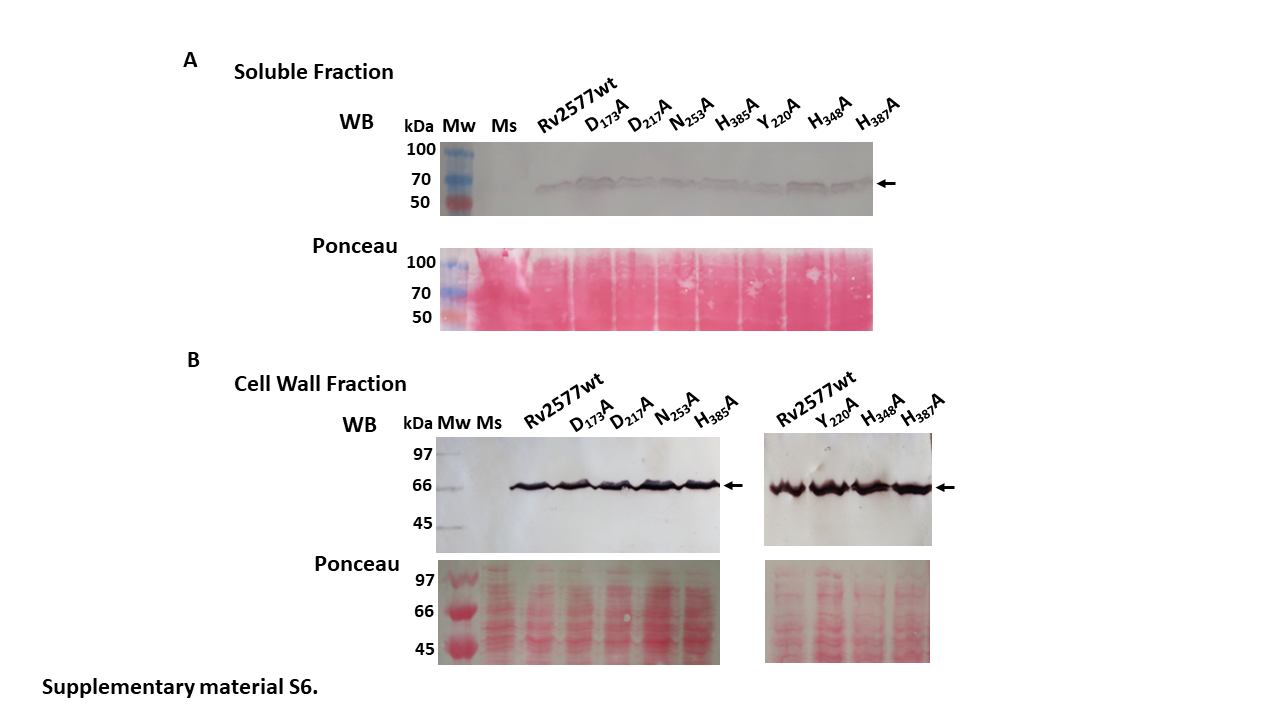

Supplement: Supplementary Figure 5 — Expression of Rv2577 wild type and site-directed mutants in M. smegmatis. Western blot analysis with α-HA antibody (top panel) of the soluble (inner membrane and cytoplasm) (A) and cell wall (B) fractions of proteins from M. smegmatis (Ms) and M. smegmatis overexpressing the Rv2577 wild type (Rv2577wt) or its site-directed mutants D173A, D217A, N253A, H385A, Y220A, H348A, and H387A. The arrows indicate the presence of Rv2577. The same protein amount (100 μg) was loaded per lane, as shown by Ponceau red staining (bottom panel in A) and (B). Mw: Prestained Blue plus® protein marker 14–100 kDa (TransGen Biotech) (A) or unstained LMW-SDS marker (GE Healthcare) (B). [file Image_5.TIF]

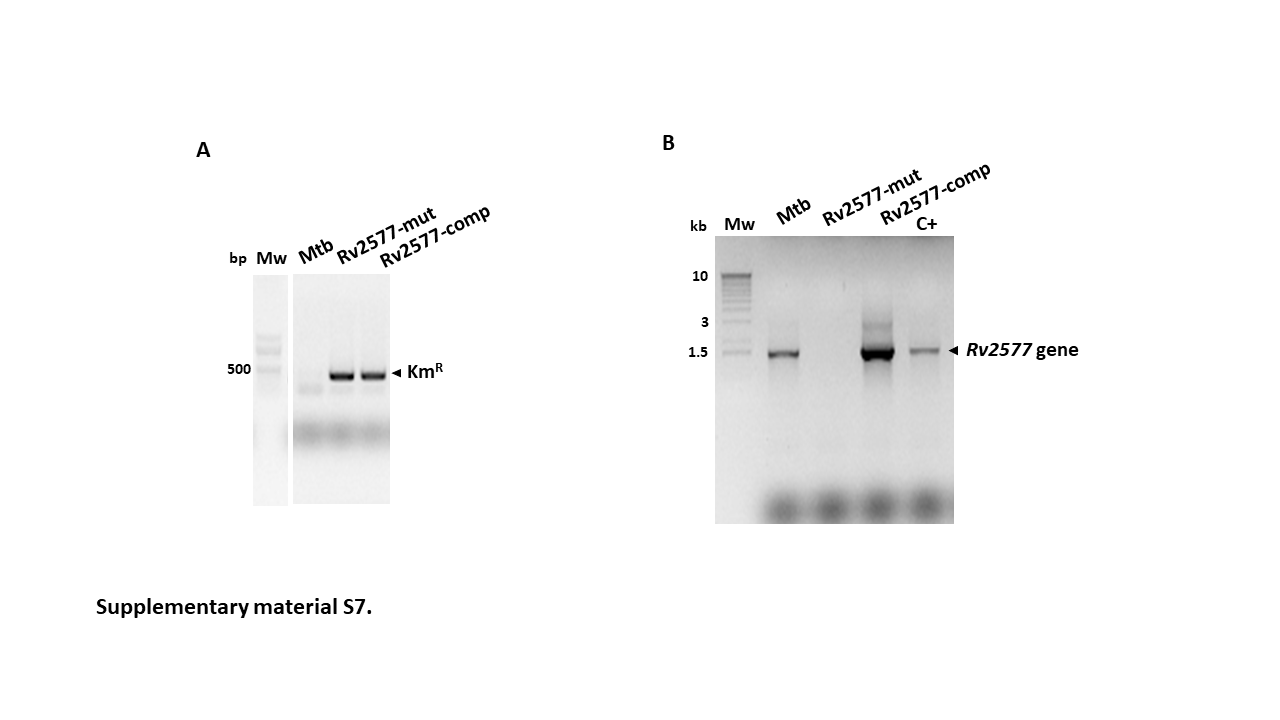

Supplement: Supplementary Figure 6 — The integrity of the Rv2577 gene in the M. tuberculosis strains. (A) PCR amplification of the kanamycin resistance (KmR) from the genomes of M. tuberculosis CDC 1551 wild type (Mtb), Rv2577 mutant (Rv2577-mut) and complemented (Rv2577-comp) strains. A4̃00 bp fragment of the KmR was amplified from the Rv2577 mutant and complemented strains (arrow). (B) PCR amplification of Rv2577 from the M. tuberculosis strains studied in A); a 1590 bp fragment corresponding to the full Rv2577 gene was amplified from the M. tuberculosis wild type and complemented strains (arrow). Mw: DNA marker 1 kb (Roche). C+ : plasmid pML2031:Rv2577 was used as a positive control. [file Image_6.TIF]

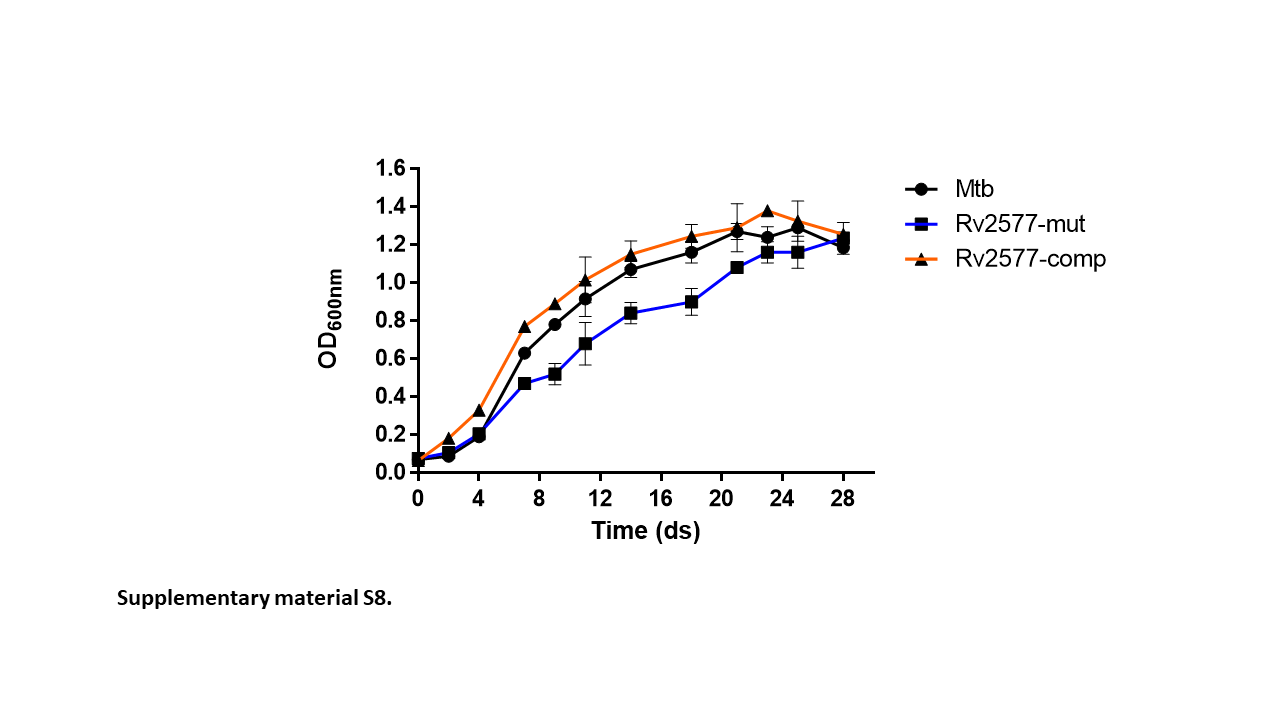

Supplement: Supplementary Figure 7 — Growth kinetics of the M. tuberculosis strains. Mtb CDC 1551 (Mtb, black), Rv2577 mutant (Rv2577-mut, blue) and complemented (Rv2577-comp, orange) strains were growth in 7H9 rich media as described in section “Materials and Methods.” The bacterial growth kinetic was monitored by OD600 nm measurement at different times (days). The data are representative of three independent experiments performed in duplicate. [file Image_7.TIF]

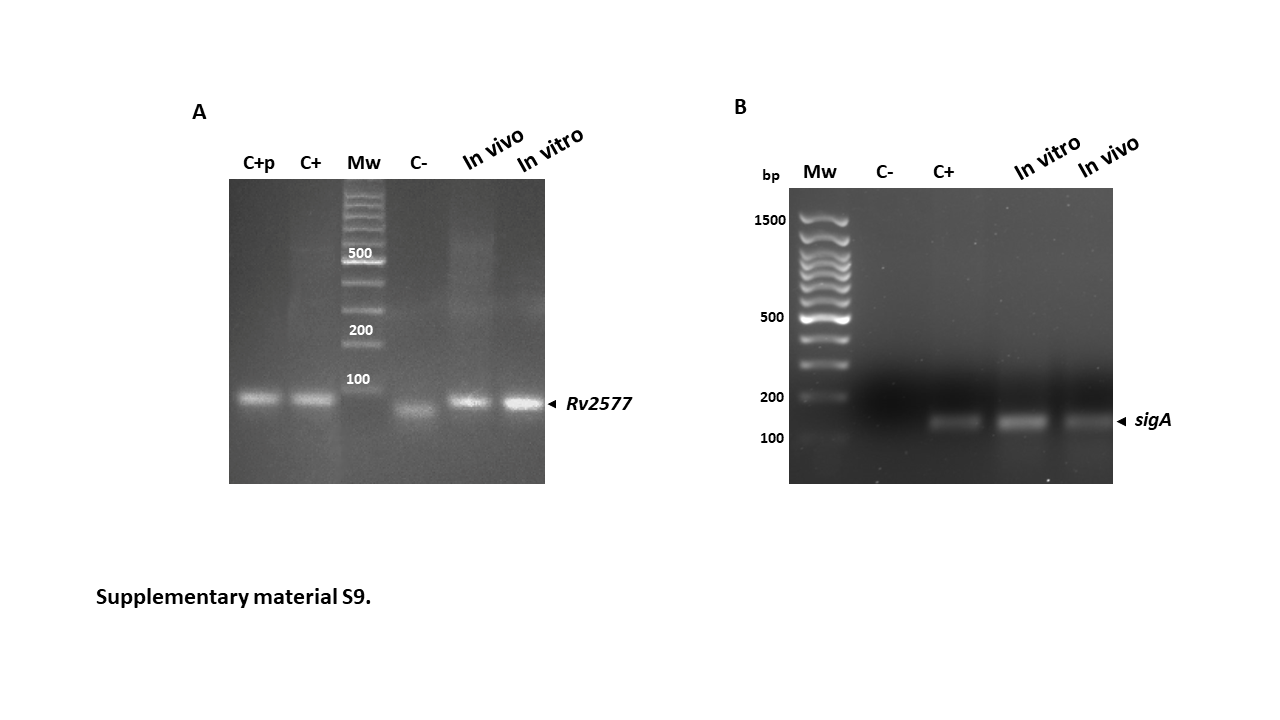

Supplement: Supplementary Figure 8 — Rv2577 gene is transcribed during the in vitro and in vivo growth of Mtb. (A) RT-PCR of Rv2577 gene in a 2% agarose gel electrophoresis. Total RNA and cDNA were obtained from Mtb CDC 1551 bacterium growing in vitro into rich media (in vitro) or during its intracellular growth after 5 h post-infection in human THP-1 macrophages (in vivo). The 81 bp fragment corresponding to the Rv2577 gene is shown by the arrow (Rv2577). The pML2031:Rv2577 vector (C+ p) and the genomic DNA from Mtb CDC 1551 strain (C+) were used as positive controls. Water was used in the PCR mix as a negative control (C−). (B) The 110 bp PCR fragment from the housekeeping sigA gene (sigA, arrow) was used as a control of transcription. Mw: 100 bp DNA ladder (TransGene Biotech). [file Image_8.TIF]

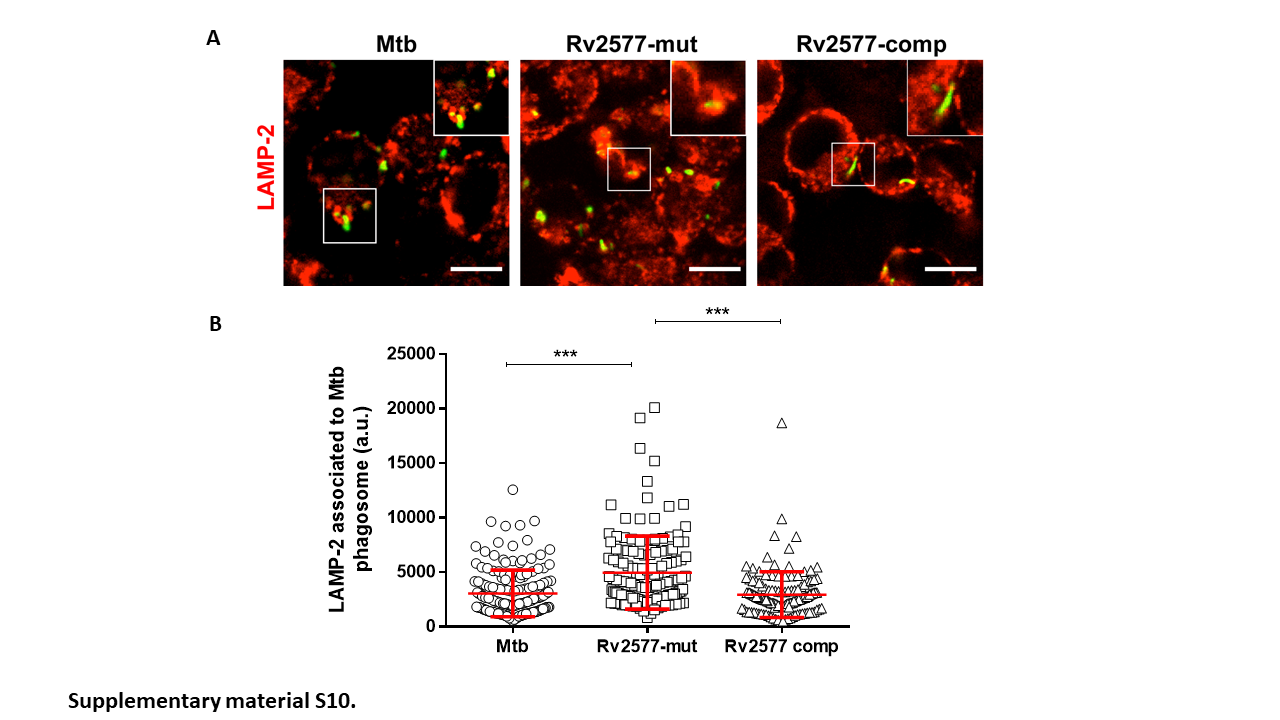

Supplement: Supplementary Figure 9 — Rv2577 participates in the arrest of murine phagosome maturation. (A) Mouse J774 macrophages were infected with M. tuberculosis wild type (Mtb) as well as the Rv2577 mutant (Rv2577-mut) and the complemented (Rv2577-comp) strains to analyze the intracellular trafficking. The bacteria were labeled with FITC (green) and LAMP-2 protein was detected using a specific antibody (red). (B) Quantification of the fluorescence observed in (A) the association of LAMP-2 to the mycobacterial compartment. The data are based on a representative experiment of three independent experiments, all performed in duplicate. ∗∗∗p < 0.001 statistical analysis using ANOVA. Number of counted phagosomes = 150 from at least 50 different macrophages. [file Image_9.TIF]

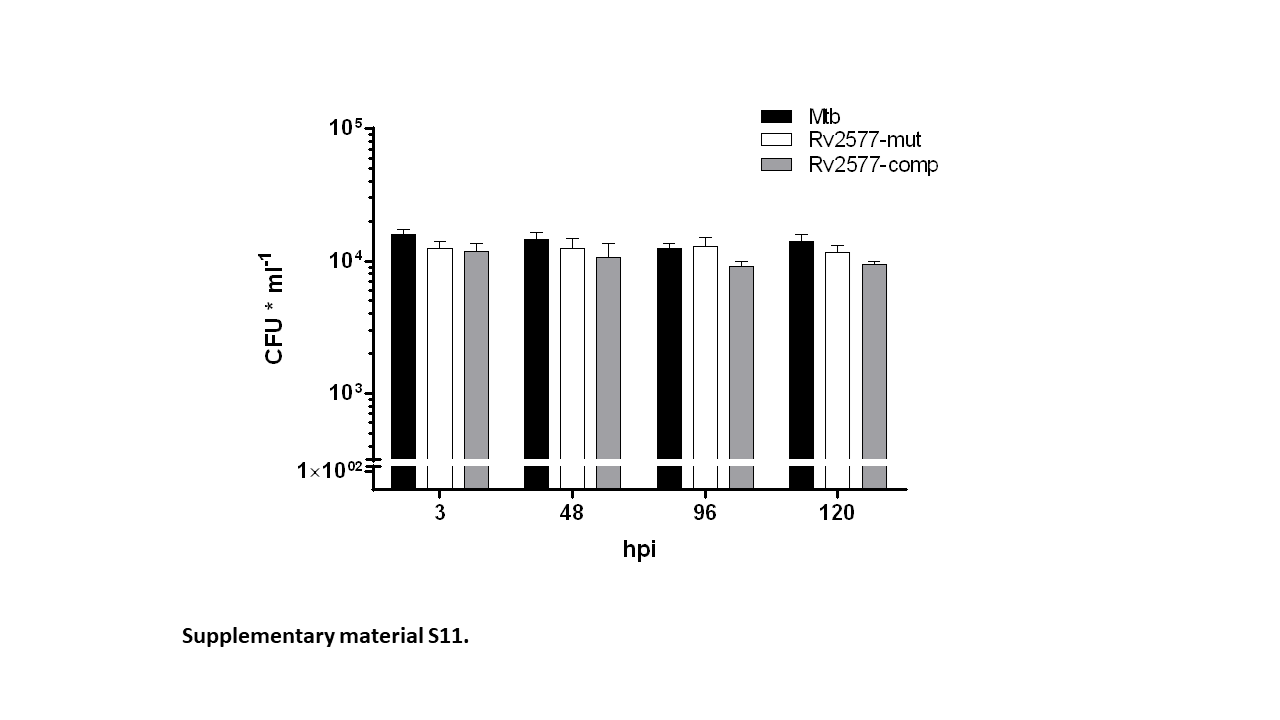

Supplement: Supplementary Figure 10 — Ex vivo survival of M. tuberculosis strains in human THP-1 macrophages. Survival of M. tuberculosis CDC 1551 wild type (Mtb), Rv2577 mutant (Rv2577-mut) and complemented (Rv2577-comp) strains measured as CFU∗ml–1 in human THP-1 macrophages after 3, 48, 96, and 120 h post-infection (hpi). Data show a representative of three experiments performed independently in duplicate. No significant differences were observed by ANOVA analysis. [file Image_10.TIF]
