# Supplementary material for: Rv2577 of Mycobacterium tuberculosis Is a Virulence Factor With Dual Phosphatase and Phosphodiesterase Functions
Source: Front Microbiol. 2020 Oct 22;11:570794. doi: 10.3389/fmicb.2020.570794 (PMC7642983; doi:10.3389/fmicb.2020.570794)
Supplement: Supplementary Table 2 — Strains, plasmids and primers used in this study. [file Table_2.DOCX]

**Supplementary Table 2. Strains, plasmids and primers used in this study.**

| Strains | Relevant description | Reference |
| --- | --- | --- |
| *M. tuberculosis* wild type | *M. tuberculosis* CDC 1551 | NR-13649. BEI Resources |
| *M. tuberculosis* Rv2577 mutant | *M. tuberculosis* CDC 1551 Rv2577::Km^R^ | NR-18473. BEI Resources |
| *M. tuberculosis* complemented | *M. tuberculosis* CDC 1551 Rv2577::Km^R^ (pVV16::Rv2577) | This study |
| *M. smegmatis* | *M. smegmatis* strain ATCC 700084 mc^2^155 | ATCC |
| Plasmids |  |  |
| pVV16 | Mycobacterial replicative vector. Km^R^ Hyg^R^ | NR-13402. BEI Resources |
| pVV16::Rv2577 | pVV16 vector carrying the Rv2577 gene in a *Nde*I restriction site. | This study |
| pML2031 | Mycobacterial replicative and expression vector. Hyg^R^ | Dr. Niederweis |
| pML2031::Rv2577wt | pML2031 carrying the Rv2577 gene into a *Nde*I/*EcoR*V restriction site. Hyg^R^ | This study |
| pML2031::Rv2577 D_173_A | pML2031::Rv2577 within a D_173_A mutation | This study |
| pML2031::Rv2577 D_217_A | pML2031::Rv2577 within a D_217_A mutation | This study |
| pML2031::Rv2577 Y_220_A | pML2031::Rv2577 within a Y_220_A mutation | This study |
| pML2031::Rv2577 N_253_A | pML2031::Rv2577 within a N_253_A mutation | This study |
| pML2031::Rv2577 H_348_A | pML2031::Rv2577 within a H_348_A mutation | This study |
| pML2031::Rv2577 H_385_A | pML2031::Rv2577 within a H_385_A mutation | This study |
| pML2031::Rv2577 H_387_A | pML2031::Rv2577 within a H_387_A mutation | This study |
| Primers |  |  |
| Rv2577wt |  | This study |
| FwRv2577Ms | 5´CATATGGGCGCCGATCTGAAGC3´ |  |
| RevRv2577Ms | 5´GATATCTCCGCCGCGCGGCTT3´ |  |
| Rv2577 D_173_A mutant |  | This study |
| D_173_A sense | 5´CACCAGCTTCGGTGCTCAGTCCACTCCCGCG3´ |  |
| D_173_A antisense | 5´CGCGGGAGTGGACTGAGCACCGAAGCTGGTG3´ |  |
| Rv2577 D_217_A mutant |  | This study |
| D_217_A sense | 5´CTGATCAACGGTGCCCTGTGTTACGCCAAC3´ |  |
| D_217_A antisense | 5´GTTGGCGTAACACAGGGCACCGTTGATCAG3´ |  |
| Rv2577 Y_220_A mutant |  | This study |
| Y_220_A sense | 5´CGGTGACCTGTGTGCCGCCAACCTGGCAC3´ |  |
| Y_220_A antisense | 5´GTGCCAGGTTGGCGGCACACAGGTCACCG3´ |  |
| Rv2577 N_253_A mutant |  | This study |
| N_253_A sense | 5´GCCGGCAGCGGGCGCTCACGAGAACGAAGTC3´ |  |
| N_253_A antisense | 5´GACTTCGTTCTCGTGAGCGCCCGCTGCCGGC3´ |  |
| Rv2577 H_348_A mutant |  | This study |
| H_348_A sense | 5´GGTCGTCTGCATGGCTCAGACCGCGATCTCC3´ |  |
| H_348_A antisense | 5´GGAGATCGCGGTCTGAGCCATGCAGACGACC3´ |  |
| Rv2577 H_385_A mutant |  | This study |
| H_385_A sense | 5´GGTGGTGTGCGGCGCCGAACACCACTACGAG3´ |  |
| D_385_A antisense | 5´CTCGTAGTGGTGTTCGGCGCCGCACACCACC3´ |  |
| Rv2577 H_387_A mutant |  | This study |
| H_387_A sense | 5´GGTGGTGTGCGGCGCCGAACACCACTACGAG3´ |  |
| D_387_A antisense | 5´CTCGTAGTGGTGTTCGGCGCCGCACACCACC3´ |  |

The *Nde*I (CATATG) and *EcoR*V (GATATC) restriction sites and the codon, which codified for each amino acid residues in the catalytic site of Rv2577 mutated by alanine, are underline.
